# Multimorbidity clusters and their associations with health-related quality of life in two UK cohorts

**DOI:** 10.1186/s12916-024-03811-3

**Published:** 2025-01-08

**Authors:** Lewis Steell, Stefanie J. Krauth, Sayem Ahmed, Grace O. Dibben, Emma McIntosh, Peter Hanlon, Jim Lewsey, Barbara I. Nicholl, David A. McAllister, Susan M. Smith, Rachael Evans, Zahira Ahmed, Sarah Dean, Colin Greaves, Shaun Barber, Patrick Doherty, Nikki Gardiner, Tracy Ibbotson, Kate Jolly, Paula Ormandy, Sharon A. Simpson, Rod S. Taylor, Sally J. Singh, Frances S. Mair, Bhautesh D. Jani, the PERFORM research team

**Affiliations:** 1 General Practice and Primary Care, School of Health and Wellbeing, University of Glasgow, Glasgow, UK; 2 AGE Research Group, Translational and Clinical Research Institute, Faculty of Medical Sciences, Newcastle University, Newcastle Upon Tyne, UK; 3 NIHR Newcastle Biomedical Research Centre, Newcastle Upon Tyne Hospitals NHS Foundation Trust, Cumbria, Northumberland, Tyne and Wear NHS Foundation Trust and Newcastle University, Newcastle Upon Tyne, UK; 4 School of Allied and Public Health Professions, Canterbury Christ Church University, Canterbury, UK; 5 Health Economics and Health Technology Assessment, School of Health and Wellbeing, University of Glasgow, Glasgow, UK; 6 MRC/CSO Social & Public Health Sciences Unit, School of Health and Wellbeing, University of Glasgow, Glasgow, UK; 7 Discipline of Public Health and Primary Care, Trinity College Dublin, Dublin, Ireland; 8 Department of Respiratory Sciences, University of Leicester, Leicester, UK; 9 University of Exeter Medical School, Exeter, UK; 10 School of Sport, Exercise and Rehabilitation Sciences, University of Birmingham, Birmingham, UK; 11 Clinical Trials Unit, University of Leicester, Leicester, UK; 12 Department of Health Science, University of York, York, UK; 13 Department of Cardiopulmonary Rehabilitation, University Hospitals of Leicester NHS Trust, Leicester, UK; 14 Institute of Applied Health Research, University of Birmingham, Birmingham, UK; 15 School of Health and Society, University of Salford, Manchester, UK; 16 Robertson Centre for Biostatistics, School of Health and Wellbeing, University of Glasgow, Glasgow, UK

**Keywords:** Multimorbidity, Quality of life, Latent class analysis, UK Biobank

## Abstract

**Background:**

Identifying clusters of multiple long-term conditions (MLTCs), also known as multimorbidity, and their associated burden may facilitate the development of effective and cost-effective targeted healthcare strategies. This study aimed to identify clusters of MLTCs and their associations with long-term health-related quality of life (HRQoL) in two UK population-based cohorts.

**Methods:**

Age-stratified clusters of MLTCs were identified at baseline in UK Biobank (*n* = 502,363, 54.6% female) and UKHLS (*n* = 49,186, 54.8% female) using latent class analysis (LCA). LCA was applied to people who self-reported ≥ 2 LTCs (from *n* = 43 LTCs [UK Biobank], *n* = 13 LTCs [UKHLS]) at baseline, across four age-strata: 18–36, 37–54, 55–73, and 74 + years. Associations between MLTC clusters and HRQoL were investigated using tobit regression and compared to associations between MLTC counts and HRQoL. For HRQoL, we extracted EQ-5D index data from UK Biobank. In UKHLS, SF-12 data were extracted and mapped to EQ-5D index scores using a standard preference-based algorithm. HRQoL data were collected at median 5 (UKHLS) and 10 (UK Biobank) years follow-up. Analyses were adjusted for available sociodemographic and lifestyle covariates.

**Results:**

LCA identified 9 MLTC clusters in UK Biobank and 15 MLTC clusters in UKHLS. Clusters centred around pulmonary and cardiometabolic LTCs were common across all age groups. Hypertension was prominent across clusters in all ages, while depression featured in younger groups and painful conditions/arthritis were common in clusters from middle-age onwards. MLTC clusters showed different associations with HRQoL. In UK Biobank, clusters with high prevalence of painful conditions were consistently associated with the largest deficits in HRQoL. In UKHLS, clusters of cardiometabolic disease had the lowest HRQoL. Notably, negative associations between MLTC clusters containing painful conditions and HRQoL remained significant even after adjusting for number of LTCs.

**Conclusions:**

While higher LTC counts remain important, we have shown that MLTC cluster types also have an impact on HRQoL. Health service delivery planning and future intervention design and risk assessment of people with MLTCs should consider both LTC counts and MLTC clusters to better meet the needs of specific populations.

**Supplementary Information:**

The online version contains supplementary material available at 10.1186/s12916-024-03811-3.

## Background

People living with multiple long-term conditions (MLTCs; or ‘multimorbidity’), typically defined as the co-existence of 2 or more chronic conditions, is an area of major international public health concern [1, 2]. Improved survival of people living with LTCs, coupled with increasing life expectancies and generational shifts in lifestyle behaviours, such as diets and physical activity, have facilitated the accumulation of MLTCs in populations worldwide. In the UK, approximately 20–40% of adults live with MLTCs, rising to over half of people aged > 65 years, depending on the definition applied [3–7]. In addition, people living in socio-economically deprived populations experience the onset of MLTCs up to two decades earlier than those from the least deprived areas [5, 6]. Despite the burgeoning clinical and economic burden of MLTCs [8, 9], healthcare services are almost universally designed around the treatment of single conditions and are, therefore, inadequate to meet the more complex needs of people living with MLTCs. Consequently, people with MLTCs experience fragmented healthcare, high treatment burden, increased healthcare utilisation, and higher risk of premature mortality [10–12].

The current paradigm of classifying MLTCs based on counts of co-existing conditions lacks granularity and fails to account for heterogeneity in morbidity profiles across diverse populations with MLTCs, and thus may have limited impact on policy and practice. Appeals have been made instead to identify clusters of MLTCs that commonly co-exist, to improve design of new healthcare strategies [1, 13]. There is mounting evidence that a higher number of co-existing LTCs are associated with poorer health outcomes, including increased mortality and unscheduled hospitalisations (particularly in those with ≥ 4 LTCs) [10, 14, 15]. However, there remains limited understanding of the role played by accumulation of different types of LTCs in MLTC clusters that may drive adverse health outcomes through potentially shared mechanisms. Recently, investigators have adopted novel ways of clustering MLTCs, applying data-driven approaches such as latent class analysis (LCA) to population and healthcare datasets [7, 16, 17]. There is a need, however, to validate the existence of MLTC clusters across populations, particularly as different clustering approaches have been found to identify divergent MLTC clusters even in the same dataset [17, 18]. Replicable MLTC clusters including cardiometabolic and mental health conditions have been consistently reported across studies [19] but validation of other clinically important MLTC clusters is required to advance healthcare strategies.

Improving health-related quality of life (HRQoL) is a key target for interventions and clinical management in people living with MLTCs [20, 21]. The negative association between MLTCs and HRQoL is widely recognised and a decrease of approximately − 4% (95%CI − 5.4%, − 2.4%) in HRQoL per additional LTC was reported in a meta-analysis of observational studies [22]. There is, however, very limited evidence of the association between MLTC clusters and long-term HRQoL. Elucidating the associations between MLTC clusters and HRQoL may improve understanding of underlying mechanisms, identify targets for future interventions and service developments, and inform intervention design to enhance the likelihood of improving HRQoL in specific subgroups of people with MLTCs. The aims of this study were, therefore, to identify clusters of MLTCs using LCA in two independent, population-based cohorts, and to investigate the associations between these MLTC clusters and long-term HRQoL and compare it with the association between LTC counts and long-term HRQOL.

## Methods

### Data sources

UK Biobank is a population-based cohort study that recruited approximately half a million (*n* = 502,640) community participants (aged 37–73 years at baseline) in England, Scotland, and Wales (5% response rate). Eligible participants lived within 25 miles of a participating assessment centre and were registered with a general practitioner. Baseline assessments were conducted at the time of recruitment between 2006 and 2010, where participants completed health questionnaires, interviews, and physical measures, and provided biological samples. After removing individuals who withdrew consent, our baseline analyses included 502,363 participants. A flow diagram representing participant inclusion at each stage of analysis for UK Biobank is available in Additional File 1: Fig. S1.

Understanding Society: the UK Household Longitudinal Study (UKHLS) is a household panel study that annually surveys approximately 40,000 UK households, selected to be representative of the UK general population (57.3% household response rate at baseline). Baseline surveys were conducted between 2009 and 2011. Surveys were conducted by interviewers attending participants’ home addresses or by telephone and all members of participating households were surveyed. Our analyses included only participants aged ≥ 18 years at baseline assessment (*n* = 49,186). A flow diagram representing participant inclusion at each stage of analysis for UKHLS is available in Additional File 1: Fig. S2.

### Assessment of multiple long-term conditions, sociodemographic, and lifestyle information

Health status was self-reported at baseline in both cohorts using different methods. UK Biobank participants self-reported LTCs at baseline interviews, which were subsequently collated and coded into the database by trained research nurses. LTCs were only coded if self-reported and we assume non-reporting of LTCs to imply their absence. To our knowledge, all UK Biobank participants completed this phase of baseline interviews as UK Biobank do not imply any missing data for self-reported LTCs. Our assessment of MLTCs in UK Biobank included 43 LTCs, based on a consensus-based list commonly applied in multimorbidity research [6]. Some included LTCs (e.g. painful conditions) are composite measures defined using several individually reported LTCs from UK Biobank baseline assessment, an approach widely adopted in previous research [10, 15, 23, 24] (details available in Additional File 1: Table S1). In UKHLS, participants’ self-reported LTCs were limited to a pre-specified list of 17 conditions, captured in response to the question “*Has a doctor or other health professional ever told you that you have any of these conditions?*”. Again, we assume non-reporting of LTCs to imply their absence and this question had a total of 0.26% (*n* = 127) missing data. Missing data were included in the analyses as having no self-reported LTCs. Where appropriate, some LTCs were combined to map onto the longer list used in UK Biobank; therefore, assessment of MLTCs in UKHLS was calculated using 13 LTCs (Additional File 1: Table S1). The within-cohort prevalence of individual LTCs is available in Additional File 1: Table S2. The number of LTCs reported by each participant was counted and those who reported 2 or more LTCs were classed as having multimorbidity (i.e. MLTCs) in subsequent analyses. Those who reported zero or one LTC were classed as not having MLTCs (i.e. ‘no multimorbidity’) and formed the reference group.

We extracted data on age, sex, and ethnicity from baseline questionnaires. Area level socioeconomic deprivation was measured using Townsend scores in UK Biobank [25] and nation-specific (England, Scotland, Wales, Northern Ireland) index of Multiple Deprivation (IMD) scores in UKHLS, both of which were categorised into quintiles. Body mass index (BMI) (kg/m^2^) was derived from physical measurements in UK Biobank and from self-reported height and weight in UKHLS. Smoking status was categorised as current, previous, or never in both datasets. Alcohol frequency (self-reported) was included as a categorical variable in both datasets and weekly alcohol intake (units) was also included as a continuous variable in UK Biobank. Physical activity (PA) for the previous 4 weeks was categorised as none (no PA), low (light DIY only), moderate (walking for pleasure, heavy DIY, or sporting activity), or high (strenuous sporting activity) in UK Biobank [26]. UKHLS did not collect data on PA levels. Instead, the number of days participants had walked at least 30 min across the previous 4 weeks was counted and used as a continuous measure of PA levels (range 0–28). In UKHLS, data on smoking, alcohol, and PA were extracted from 1-year follow-up surveys (‘Wave B’), as these data were not collected at baseline. Frailty phenotype was calculated at baseline in UK Biobank participants and categorised as robust, pre-frail or frail according to five input criteria (low physical activity, grip strength, weight loss, exhaustion, and walking pace) [27]. The proportion of UK Biobank participants who died prior to collection of HRQoL follow-up data (defined as 01 January 2019) were also calculated for each of the identified MLTC clusters and ‘no multimorbidity’ groups. Mortality data were not available for UKHLS.

### Health-related quality of life (outcome)

The primary outcome of interest was HRQoL, assessed using EQ-5D index scores. In UK Biobank, EQ-5D index scores were derived from the EuroQol 5-dimension 5-level quality of life instrument (EQ-5D-5L), administered as part of an online only follow-up approximately 10 years after baseline assessments (*n* = 167,185). EQ-5D-5L assesses HRQoL across five domains (self-care, mobility, usual activities, pain and discomfort, and anxiety and depression) and each domain assessed across five levels of severity (no problems to extreme problems). Responses are transformed into a single score using country-specific value sets to generate the EQ-5D index—a weighted, preference-based value for HRQoL [28]. Reporting of no problems across the five domains generates the maximum EQ-5D index of 1, representing full health. An EQ-5D index of 0 represents health states equivalent to death, and negative values are health states considered worse than death. Following guidance from the National Institute for Health and Care Excellence (NICE) [29], EQ-5D-5L responses were mapped onto the EQ-5D-3L UK value set using crosswalk functions for the derivation of EQ-5D scores [30]. In this UK value set, the lowest EQ-5D index score was − 0.594. EQ-5D-5L was not collected at baseline in UK Biobank; therefore, self-rated health (excellent, good, fair, poor) was used as an alternative measure of self-reported heath status at baseline in this cohort.

In UKHLS, HRQoL was assessed using the generic 12-item short form health survey (SF-12) [31]. SF-12 evaluates health across eight domains: physical function, limitations due to physical problems, pain, general health, vitality, social functioning, limitations due to emotional problems, and mental health. SF-12 data were subsequently mapped onto EQ-5D index scores using the methods of Gray et al. [32] prior to analyses. Our analyses used HRQoL data that was collected during the approximately 5 years follow-up visit from baseline (‘Wave F’ of UKHLS data collection) (*n* = 21,837).

### Statistical analyses

#### Latent class analysis (LCA)

Firstly, we applied LCA to both datasets to identify clusters of MLTCs. These analyses included only participants with two or more self-reported LTCs at baseline. In summary, LCA identifies unobserved subgroups (i.e. ‘latent classes’) within a population based on known indicator variables. Here, the indicators were self-reported single-condition LTCs (UK Biobank *n* = 43, UKHLS *n* = 13). LTCs were treated as present if self-reported, or otherwise absent, meaning LCA models had no missing data. LCA was applied separately to four age categories to account for age-related differences in morbidity across the adult lifespan: 18–36 years (UKHLS only), 37–54 years (both datasets), 55–73 years (both datasets), 74 + years (UKHLS only). In each age group/dataset combination, we explored LCA models containing 1–10 latent classes. Optimal LCA model selection was based on several indices, including model parsimony (Bayesian Information Criterion [BIC] & sample size adjusted BIC), stability, classification (entropy), and clinical interpretability of the identified classes [33, 34]. Individuals were assigned to the latent class for which they had the highest posterior probability of belonging. Classes were labelled according to within and between cluster probabilities/prevalence of LTCs. A detailed description of LCA methods and model class solutions is available elsewhere [12]. As MLTC is strongly associated with social determinants of health [35], we tested the influence of socioeconomic status (SES) on MLTC clusters as a sensitivity analysis. LCA was repeated in each of the subgroups, across the two cohorts, with SES included as an additional indicator variable. The summary characteristics of MLTC clusters derived with SES as an indicator were then compared to those derived in the main LCA analysis (i.e. without SES).

#### Association between MLTCs and HRQoL

We then investigated associations between MLTCs and HRQoL index scores using tobit regression models with robust standard errors. Tobit regression assumes the existence of a continuous latent outcome and has been extensively applied to HRQoL research to account for the ceiling effect observed in indexes such as EQ-5D [36]. HRQoL data were collected at median 10 (IQR 9.3, 10.7) years follow-up in UK Biobank and 5 (IQR 4.5, 6.1) years follow-up in UKHLS. Firstly, we investigated the associations between MLTCs and HRQoL using LTC counts as a categorical predictor variable using the following classes: No multimorbidity (reference group), 2 LTCs, 3 LTCs, or ≥ 4 LTCs (Model 1). Next, using the LTC clusters identified during LCA, we assessed the relationship between clusters and HRQoL, again using the ‘no multimorbidity’ population as the reference group (Model 2). All models were adjusted for available sociodemographic covariates, including age (as a continuous variable in years), sex, ethnicity, deprivation (Townsend [UK Biobank] or IMD [UKHLS] quintile), BMI, smoking, physical activity, alcohol frequency, alcohol weekly units (UK Biobank only), frailty phenotype (UK Biobank only), and baseline HRQoL (self-rated health [UK Biobank] and EQ-5D index [UKHLS]). A final combined model including LTC counts (continuous) and MLTC clusters was conducted to investigate the relationship of HRQoL with cluster membership controlling for number of self-reported LTCs (Model 3). Non-linear associations between LTC count and EQ-5D index were formally assessed using fractional polynomials; however, no deviation from a linear relationship was observed, so LTC count was included only as a continuous covariate in these combined models. As a sensitivity analysis, we conducted the same models using ordinary least squares (OLS) regression, to compare the magnitude and direction of observed associations. All regression modelling was conducted in Stata 17 (StataCorp. 2021. TX: StataCorp LLC.)

### Ethical considerations

All participants of both studies provided written consent prior to data collection. UK Biobank has ongoing approval by the NHS National Research Ethics Service (16/NW/0274). These analyses were performed as part of UK Biobank project ID 14151. UKHLS data collection was approved by the University of Essex Ethics Committee. UKHLS data [37] were distributed via the UK data service (Project ID: 221,571). This study was conducted as part of a National Institute for Health and Care Research (NIHR)-funded research programme grant (ID: NIHR202020).

## Results

### Study populations

Baseline data were available for 502,363 participants from UK Biobank and 49,186 participants from UKHLS (Table 1). Both cohorts comprised approximately 54% female participants and median age was higher in UK Biobank (58 vs 45 years). UK Biobank participants were more likely to be of white ethnicity (94% vs 77%) and to be from areas of low socioeconomic deprivation (42% vs 17% in the least deprived quintile) compared to UKHLS participants. UKHLS participants were more likely to be from areas of greatest socioeconomic deprivation than the general population (23% of participants from most deprived quintile). The proportions of people living with MLTCs at baseline were 33% (*n* = 165,123) in UK Biobank (using *n* = 43 LTCs) and 18% (*n* = 8876) in UKHLS (using *n* = 13 LTCs). Self-rated health at baseline was ‘excellent’ or ‘good’ in 74% of UK Biobank participants. In UKHLS, at baseline, mapped median EQ-5D index was 0.848 (IQR 0.727, 1) and approximately 40% of respondents scored the maximum index value of 1 (i.e. ‘full health’). The within-cohort prevalence of each included single condition LTC is available in Additional File 1: Table S2.

**Table 1:** Summary baseline cohort characteristics in UK Biobank and UKHLS

	**UK Biobank** **(*n* = 502,363)**	**UKHLS** **(*n* = 49,186)**
Age (years), median (IQR)	58 (50, 63)	45 (32, 60)
Age category, *n* (%)		
18–36 years		16,105 (32.7)
37–54 years	194,109 (38.6)	16,726 (34)
55–73 years	308,254 (61.4)	12,308 (25)
74 + years		4047 (8.2)
Female sex, *n* (%)	273,299 (54.4)	28,857 (54.6)
White ethnicity, *n* (%)	472,569 (94.6)	37,950 (77.2)
Socioeconomic deprivation ^a^, *n* (%)		
Quintile 1 (Most deprived)	77,265 (15.4)	11,462 (23.3)
2	77,851 (15.5)	10,283 (20.9)
3	65,816 (13.1)	9659 (19.6)
4	67,720 (13.5)	9057 (18.4)
Quintile 5 (Least deprived)	213,088 (42.4)	8725 (17.7)
Missing	623 (0.1)	0 (0)
Number of long-term conditions ^b^, *n* (%)		
0 LTCs	173,072 (34.5)	27,291 (55.5)
1 LTC	164,168 (32.7)	13,019 (26.5)
2 LTCs	95,527 (19)	5189 (10.6)
3 LTCs	43,320 (8.6)	2271 (4.6)
≥ 4 LTCs	26,276 (5.2)	1416 (2.9)
*HRQoL*		
EQ-5D index ^c^, median (IQR)		0.848 (0.727, 1)
Self-rated health, *n* (%)		
Excellent	81,832 (16.3)	
Good	288,946 (57.5)	
Fair	105,332 (21)	
Poor	22,768 (4.5)	
Missing	3485 (0.7)	

^a^Using population distribution of Townsend scores (UK Biobank) and country-specific IMD scores amalgamated into a single variable (UKHLS)

^b^Defined using self-reported LTCs from lists of *n* = 43 (UK Biobank) and *n* = 13 (UKHLS)

^c^*n* = 45,774 (~ 6.9% missing)

Detailed baseline cohort characteristics stratified by age and MLTC clusters (including ‘no multimorbidity’ groups for comparison) are available in Additional File 1: Tables S3 – S8. Briefly, all MLTC clusters displayed lower HRQoL (either self-rated health [UK Biobank] or EQ-5D index [UKHLS]) compared to the age-matched no multimorbidity reference groups. MLTC clusters were also typically associated with higher BMI, lower physical activity, and higher proportion of smokers than the no multimorbidity groups (Additional File 1: Tables S3 – S8). A comparison of cohort characteristics for participants with and without follow-up HRQoL data in each cohort are available in Additional File 1: Tables S9—S10. In both cohorts, participants who were male, of non-white ethnicity, from areas of higher socioeconomic deprivation, had lower baseline self-rated health, and higher number of co-existing LTCs were less likely to have HRQoL follow-up data.

### MLTC clusters

#### Overview

In summary, the selected LCA models included 15 MLTC clusters across four age strata in UKHLS and nine MLTC clusters across two age strata in UK Biobank.

#### 18–36 years

Three MLTC clusters were identified in this age group in UKHLS. Asthma and depression were the predominant LTCs in this age group, with asthma having 100% prevalence in two clusters (‘*Pulmonary*’ and ‘*Depression & Asthma*’ clusters) and depression having 100% prevalence in one (‘*Depression & Asthma*’) and 59% prevalence in another (‘*Cardiometabolic*’). Arthritis was also among the most prevalent LTCs in two of the three identified clusters. The cluster with 100% prevalence of co-existing depression and asthma had the fewest people but the highest proportion of females (73.8%) and people from the most socioeconomically deprived areas (38.7%).

#### 37–54 years

Five MLTC clusters were identified in each dataset for this age group, with some similarities (Table 2). A cluster centred around 100% prevalence of asthma was identified in both cohorts, but with accompanying high prevalence of COPD in UKHLS only. The ‘*Pain* + ’ (UK Biobank) and ‘*Arthritis* + ’ (UKHLS) clusters were also comparable, including a similar proportion of the population for that age group (2.6% and 2.8%, respectively). Additionally, in both datasets, a single cluster with similar within cluster prevalence of thyroid disease and cancer was observed. Despite the similarities between datasets, hypertension was among the top three most prevalent LTCs in all clusters in UKHLS, whereas it was only highly prevalent in the largest cluster in UK Biobank. The two clusters with highest hypertension prevalence in the respective cohorts also had the highest proportion of people from socioeconomically deprived areas. Coronary heart disease was the leading LTC in a single cluster in UKHLS, which also had the highest median number of LTCs (3 vs 2 for all other clusters) and the lowest proportion of females.

**Table 2:** Summary of identified clusters derived from LCA in people with MLTC

Cluster Label	Leading LTCs (within-cluster prevalence [%])	*N* (%) ^a^	No. of LTCs ^b^, median (IQR)	Female (%)	Most deprived ^c^ (%)	Mortality ^d^, *n* (%)	Predicted probability of cluster membership, mean (SD)
	***18–36 years***						
	**UKHLS (*n* = 16,105)**						
No multimorbidity		15,416 (95.7)	0 (0, 0)	54.9	29.6		
*Pulmonary*	Asthma (100%), Hypertension (33%), Arthritis (23%), **COPD** (14%)	270 (1.7)	2 (2, 2)	65.2	30.0		0.95 (0.07)
*Cardiometabolic*	Depression (59%), **Hypertension** (55%), **Diabetes** (23%), Arthritis (21%)	251 (1.6)	2 (2, 2)	69.7	35.5		0.99 (0.07)
*Depression & Asthma*	**Depression** (100%), Asthma (100%)	168 (1.0)	2 (2, 3)	73.8	38.7		0.85 (0.13)
	***37–54 years***						
	**UK BIOBANK (*n* = 194,109)**						
No multimorbidity		151,975 (78.3)	0 (0, 1)	54.6	16.7	2170 (1.4)	
*Hypertension & Diabetes*	**Hypertension** (100%), **Diabetes** (22%), Painful conditions (22%)	16,126 (8.3)	2 (2, 3)	47.1	25.4	728 (4.5)	0.82 (0.16)
*Asthma* +	**Asthma** (100%), Painful conditions (24%), Psoriasis or eczema (19%)	9680 (5.0)	2 (2,3)	63.8	21.4	247 (2.5)	0.90 (0.16)
*Thyroid & Cancer*	**Thyroid disease** (24%), Dyspepsia (24%), **Cancer** (22%)	6006 (3.1)	2 (2, 2)	70.0	21.3	270 (4.5)	0.99 (0.05
*Depression & Anxiety*	**Depression** (100%), Painful conditions (25%), **Anxiety** (19%)	5250 (2.7)	2 (2, 3)	70.5	27.1	173 (3.3)	0.82 (0.15)
*Pain* +	**Painful conditions** (100%), Dyspepsia (21%), Hypertension (17%)	5072 (2.6)	2 (2, 3)	64.2	22.0	131 (2.6)	0.97 (0.11)
	**UKHLS (*n* = 16,726)**						
No multimorbidity		14,542 (86.9)	0 (0, 1)	53.4	21.2		
*Pulmonary*	**Asthma** (100%), Depression (36%), Hypertension (35%), **COPD** (18%)	805 (4.8)	2 (2, 3)	68.7	30.1		0.99 (0.03)
*Depression, Thyroid & Cancer*	Hypertension (62%), **Depression** (58%), **Thyroid disease** (25%), **Cancer** (20%)	485 (2.9)	2 (2, 2)	65.0	25.0		0.99 (0.03)
*Arthritis* +	**Arthritis** (100%), Hypertension (50%), Depression (38%)	466 (2.8)	2 (2, 3)	67.2	32.8		0.99 (0.04)
*Cardiovascular*	**CHD** (100%), Hypertension (57%), Arthritis (38%), Asthma (26%), Diabetes (21%), Stroke (11%)	218 (1.3)	3 (2, 4)	45.0	42.2		0.92 (0.11)
*Hypertension & Diabetes*	**Hypertension** (100%), **Diabetes** (100%)	210 (1.3)	2 (2, 3)	51.9	44.8		0.81 (0.16)
	***55–73 years***						
	**UK BIOBANK (*n* = 308,254)**						
No multimorbidity		185,265 (60.1)	1 (0, 1)	53.3	11.5	9095 (4.9)	
*Cardiometabolic*	**Hypertension** (100%), Painful conditions (33%), **Diabetes** (28%), **CHD** (23%), **Stroke** (8%)	52,740 (17.1)	2 (2, 3)	41.7	18.6	5827 (11.1)	0.81 (0.14)
*Pain* +	**Painful conditions** (51%), Hypertension (34%), **Dyspepsia** (29%)	33,165 (10.8)	2 (2, 3)	66.0	15.4	2181 (6.6)	0.81 (0.14)
*Pulmonary*	**Asthma** (100%), Hypertension (36%), Painful conditions (29%), **COPD** (12%)	20,624 (6.7)	2 (2, 3)	60.7	15.5	1562 (7.6)	0.68 (0.15)
*Cancer* +	**Cancer** (100%), Hypertension (44%), Painful conditions (27%)	16,460 (5.3)	2 (2, 3)	62.3	13.8	2115 (12.9)	0.79 (0.12)
	**UKHLS (*n* = 12,308)**						
No multimorbidity		8191 (66.6)	0 (0, 1)	51.5	15.4		
*Hypertension* +	**Hypertension** (100%), Arthritis (54%), Diabetes (31%)	2162 (17.6)	2 (2, 3)	55.4	22.0		0.88 (0.15)
*Pulmonary*	**Asthma** (100%), Arthritis (58%), Hypertension (34%), **COPD** (32%)	730 (5.9)	2 (2, 2)	65.5	20.8		0.81 (0.21)
*Depression, Thyroid & Cancer*	Arthritis (78%), **Depression** (30%), **Thyroid disease** (28%), **Cancer** (27%)	641 (5.2)	3 (2, 4)	68.0	17.6		0.94 (0.07)
*Cardiovascular*	**CHD** (82%), Hypertension (52%), Arthritis (48%), Diabetes (33%), **Stroke** (19%)	584 (4.7)	3 (2, 4)	38.7	30.3		0.77 (0.16)
	***74* + *years***						
	**UKHLS (*n* = 4047)**						
No multimorbidity		2161 (53.4)	1 (0, 1)	52.3	15.8		
*Arthritis* +	**Arthritis** (100%)**,** Hypertension (70%), CHD (31%)	959 (23.7)	3 (2, 3)	66.0	18.4		1.00 (0.00)
*Cardiometabolic*	Hypertension (78%), **CHD** (49%), **Diabetes** (40%) **Stroke** (22%)	547 (13.5)	2 (2, 3)	42.1	19.7		1.00 (0.00)
*Pulmonary*	**Asthma** (100%), Arthritis (59%), Hypertension (55%), CHD (29%), **COPD** (24%)	380 (9.4)	3 (2, 4)	61.1	22.4		1.00 (0.00)

Clusters identified from LCA in people living with MLTCs. ‘No multimorbidity’ group added for reference. Leading LTCs were defined as the most prevalent 2–3 LTCs within the cluster. Bolded LTCs are over-represented LTCs with higher than expected within and between-cluster prevalence

^a^*N* (%) from age group assigned to each cluster (including ‘No multimorbidity’ population added for reference)

^b^From *n* = 43 LTCs (UK Biobank) or *n* = 13 LTCs (UKHLS)

^c^% in cluster from most deprived 20% of UK population based on Townsend scores (UK Biobank [< 0.5% missing data]) or IMD scores (UKHLS [no missing data])

^d^*N* (%) from each group that died before start of online follow up in UK Biobank only (defined as 1st January 2019). Mortality data were not available for UKHLS

#### 55–73 years

Four MLTC clusters were identified in each cohort for this age group (Table 2). Painful conditions and arthritis (used in the definition of ‘painful conditions’) were prominent across all clusters in UK Biobank and UKHLS, respectively, and hypertension featured in all but one cluster (‘*Arthritis, depression and cancer*’ [UKHLS]). One cluster of combined cardiovascular and metabolic diseases was identified in each cohort, high within prevalence of similar LTCs (hypertension, pain/arthritis, CHD, diabetes, and stroke). This was the largest identified cluster in UK Biobank and was characterised by 100% prevalence of hypertension (labelled ‘*Cardiometabolic*’), whereas in UKHLS it was the smallest cluster and dominated by CHD (82% prevalence) (labelled ‘*Cardiovascular*’). These clusters were again distinct for having the lowest proportion of female participants and highest proportion of socioeconomic deprivation of all groups for that age. The largest cluster in UKHLS (‘*Hypertension* + ’) had the same three most prevalent conditions to the largest cluster in UK Biobank (hypertension, pain/arthritis, diabetes) but with lower prevalence of accompanying cardiovascular diseases.

#### 74 + years

Three MLTC clusters were identified in this age group in UKHLS (Table 2). These clusters had very similar profiles to three of the identified clusters in the 37–54 and 55–73 years age groups for UKHLS. One cluster centred around pulmonary conditions, one inclusive of cardiometabolic conditions, and another with high prevalence of arthritis.

In sensitivity analyses, when SES was included as an additional indicator variable in LCA models, the composition of MLTC clusters was broadly similar in the UK Biobank cohort and almost unchanged in the UKHLS cohort (please see Additional File 1: Table S11).

### Associations of MLTCs with HRQoL

Tobit regression results for UK Biobank and UKHLS are presented in Tables 3 and 4, respectively. After excluding participants with missing covariates, a total of 163,121 and 16,819 participants were included in regression analyses in UK Biobank and UKHLS, respectively (Additional File 1: Figures S1—S2). EQ-5D index data were collected at median 10 years (IQR 9.3, 10.7) follow-up in UK Biobank and 5 years (IQR: 4.5, 6.1) follow-up in UKHLS. An inverse association between LTC counts and EQ-5D index at follow-up was observed in both datasets in the 37–54 and 55–73 years age groups, with the ≥ 4 LTCs group having the greatest deficit compared to the ‘no multimorbidity’ group (Tables 3 and 4, Model 1). No association was observed between ≥ 4 LTCs and EQ-5D index in the youngest group for UKHLS, due to extremely small numbers with ≥ 4 LTCs in this age group (*n* = 6).

**Table 3:** Regression results for EQ-5D index scores at median 10-year follow up in UK Biobank

Model 1: LTC Counts					Model 2: MLTC Clusters				
	***N***	***β* (95% CI)**	**Std. Error**	***p*-value**		***N***	***β* (95% CI)**	**Std. Error**	***p*-value**
***37–54 years***									
No multimorbidity	54,798	Ref			No multimorbidity	54,798	Ref		
2 LTCs	8949	− 0.044 (− 0.049, − 0.039)	0.003	< 0.001	*Hypertension & Diabetes*	4405	− 0.034 (− 0.042, − 0.027)	0.004	< 0.001
3 LTCs	2936	− 0.073 (− 0.082, − 0.065)	0.004	< 0.001	*Asthma* +	3455	− 0.056 (− 0.064, − 0.049)	0.004	< 0.001
≥ 4 LTCs	1340	− 0.119 (− 0.132, − 0.106)	0.007	< 0.001	*Thyroid & Cancer*	2030	− 0.037 (− 0.046, − 0.027)	0.005	< 0.001
					*Depression & Anxiety*	1619	− 0.101, (− 0.112, − 0.090)	0.006	< 0.001
					*Pain* +	1716	− 0.092 (− 0.101, − 0.082)	0.005	< 0.001
***55–73 years***									
No multimorbidity	63,694	Ref			No multimorbidity	63,694	Ref		
2 LTCs	19,153	− 0.036 (− 0.039, − 0.032)	0.002	< 0.001	*Cardiometabolic*	11,765	− 0.036 (− 0.041, − 0.032)	0.002	< 0.001
3 LTCs	8049	− 0.058 (− 0.062, − 0.053)	0.002	< 0.001	*Pain* +	9267	− 0.069 (− 0.074, − 0.065)	0.002	< 0.001
≥ 4 LTCs	4202	− 0.089 (− 0.096, − 0.082)	0.003	< 0.001	*Pulmonary*	6039	− 0.041 (− 0.046, − 0.035)	0.003	< 0.001
					*Cancer* +	4333	− 0.032 (− 0.038, − 0.026)	0.003	< 0.001

Covariates: age, sex, ethnicity, deprivation (Townsend quintile), BMI, smoking, alcohol frequency, alcohol weekly units, physical activity, frailty phenotype, and baseline self-rated health

**Table 4:** Regression results for EQ-5D index scores at median 5-year follow-up in UKHLS

	**Model 1: LTC Counts**				**Model 2: MLTC Clusters**				
**LTC Count**	***N***	***β* (95% CI)**	**Std. Error**	***p*-value**	**MLTC cluster**	***N***	***β* (95% CI)**	**Std. Error**	***p*-value**
***18–36 years***									
No multimorbidity	3946	Ref			No multimorbidity	3946	Ref		
2 LTCs	166	− 0.086 (− 0.129, − 0.044)	0.022	< 0.001	*Pulmonary*	86	− 0.082 (− 0.140, − 0.024)	0.030	0.01
3 LTCs	38	− 0.179 (− 0.257, − 0.101)	0.040	< 0.001	*Cardiometabolic*	79	− 0.131 (− 0.201, − 0.061)	0.036	< 0.001
≥ 4 LTCs	6	− 0.154 (− 0.529, 0.221)	0.191	0.42	*Depression & Asthma*	45	− 0.105 (− 0.162, − 0.048)	0.029	< 0.001
***37–54 years***									
No multimorbidity	5486	Ref			No multimorbidity	5486	Ref		
2 LTCs	565	− 0.106 (− 0.134, − 0.078)	0.014	< 0.001	*Pulmonary*	327	− 0.128 (− 0.163, − 0.093)	0.018	< 0.001
3 LTCs	188	− 0.157 (− 0.203, − 0.112)	0.023	< 0.001	*Depression, Thyroid & Cancer*	203	− 0.071 (− 0.117, − 0.026)	0.023	0.002
≥ 4 LTCs	89	− 0.283 (− 0.351, − 0.215)	0.035	< 0.001	*Arthritis* +	183	− 0.167 (− 0.212, − 0.121)	0.023	< 0.001
					*Cardiovascular*	70	− 0.282 (− 0.366, − 0.199)	0.042	< 0.001
					*Hypertension & Diabetes*	59	− 0.112 (− 0.191, − 0.033)	0.040	0.01
***55–73 years***									
No multimorbidity	3621	Ref			No multimorbidity	3621	Ref		
2 LTCs	1030	− 0.060 (− 0.079, − 0.041)	0.009	< 0.001	*Hypertension* +	895	− 0.069 (− 0.090, − 0.049)	0.011	< 0.001
3 LTCs	419	− 0.091 (− 0.120, − 0.062)	0.015	< 0.001	*Pulmonary*	328	− 0.085 (− 0.116, − 0.054)	0.016	< 0.001
≥ 4 LTCs	255	− 0.181 (− 0.219, − 0.143)	0.019	< 0.001	*Depression, Thyroid & Cancer*	283	− 0.08 (− 0.114, − 0.045)	0.018	< 0.001
					*Cardiovascular*	198	− 0.134 (− 0.175, − 0.094)	0.021	< 0.001
***74* + *years***									
No multimorbidity	541	Ref			No multimorbidity	541	Ref		
2 LTCs	263	− 0.067 (− 0.110, − 0.025)	0.022	0.002	*Arthritis* +	252	− 0.094 (− 0.137, − 0.051)	0.022	< 0.001
3 LTCs	126	− 0.103 (− 0.156, − 0.049)	0.027	< 0.001	*Cardiometabolic*	138	− 0.072 (− 0.126, − 0.018)	0.027	0.01
≥ 4 LTCs	80	− 0.081 (− 0.151, − 0.011)	0.036	0.02	*Pulmonary*	79	− 0.042 (− 0.110, 0.027)	0.035	0.23

Covariates: age, sex, ethnicity, deprivation (IMD quintile), BMI, smoking, alcohol frequency, physical activity, and baseline EQ index. ‘No multimorbidity’ were the reference group in all models

All identified MLTC clusters were associated with deficits in long-term EQ-5D index scores to varying degrees, after adjusting for available sociodemographic and lifestyle factors (Tables 3 and 4, Model 2). In the younger age groups, clusters with highest prevalence of depression (UKHLS 18–36: ‘*Depression and asthma*’ and ‘*Hypertension and diabetes*’; UK Biobank 37–54 years: ‘*Depression and anxiety*’) were associated with some of the largest deficits in EQ-5D index (Tables 3 and 4). In UK Biobank, clusters with 100% prevalence of painful conditions also displayed large deficits in EQ-5D index in both age groups (Table 3). In UKHLS, two clusters of predominantly cardiovascular conditions, in the 37–54 years and 55–73 years age groups, were associated with the poorest HRQoL of any clusters when compared to the respective group with ‘no multimorbidity’ (Table 4).

In the combined models, including with both MLTC clusters and counts (Additional File 1: Tables S12—S13), the negative associations between MLTC clusters and EQ-5D index were no longer significant in most cases. In UK Biobank, two clusters with high prevalence of painful conditions (both labelled ‘*Pain* + ’) and one with mental health conditions (‘*Depression & Anxiety*’) remained negatively associated with EQ-5D index when controlling for LTC counts. In UKHLS, only the ‘*Arthritis* + ’ and ‘*Cardiovascular*’ clusters in the 37–54 years age group remained negatively associated with EQ-5D index after adjusting for LTC count. In all combined models, LTC count (as a continuous predictor) remained inversely associated EQ-5D index scores. In sensitivity analysis, the reported negative associations between LTC counts and MLTC clusters with EQ-5D index scores were repeated when using OLS regression, although observed effect sizes were generally smaller (Additional File 1: Tables S14—S15).

### Predicted HRQoL: LTC counts vs MLTC clusters

**Fig. 1:**
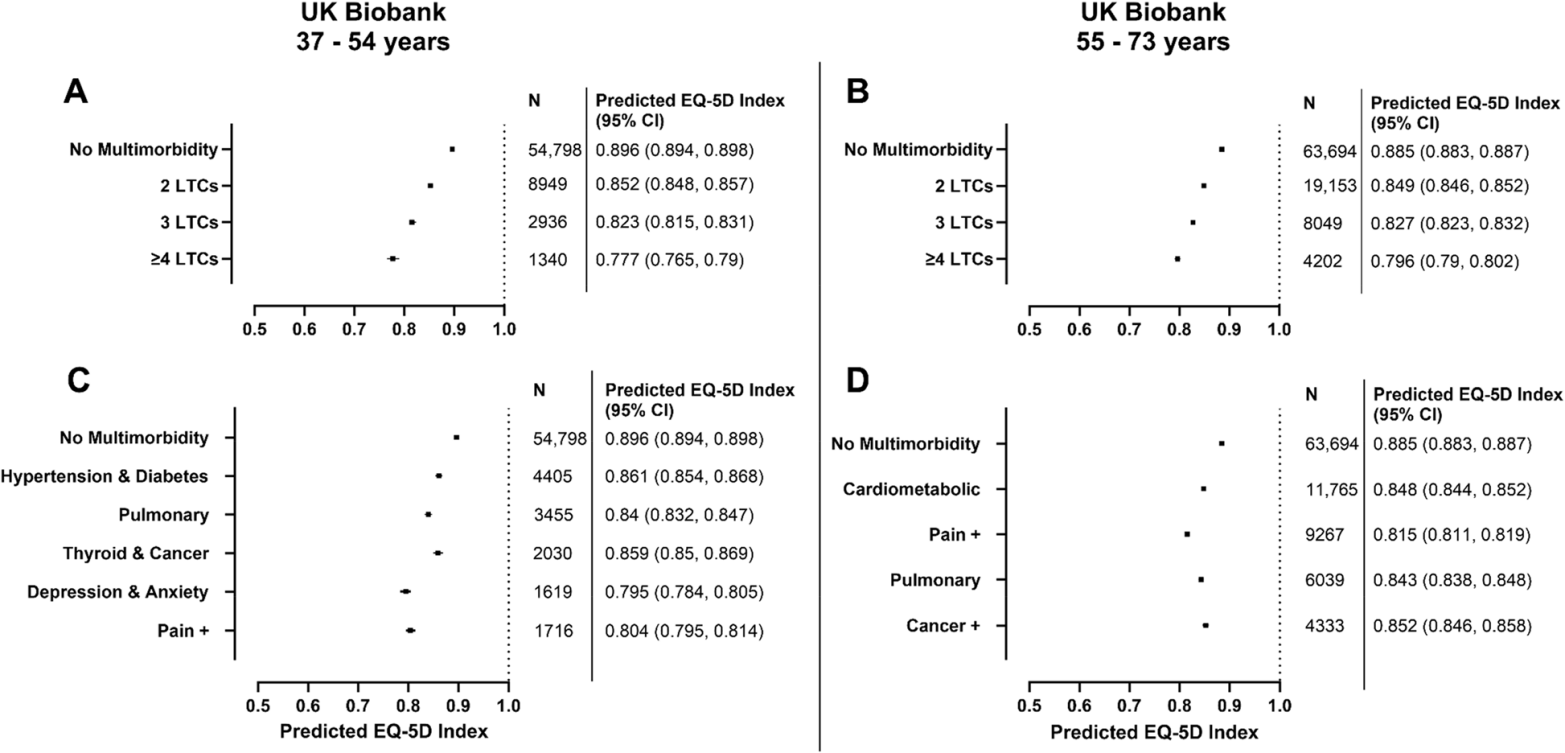
Predicted values for EQ-5D index at 10-year follow up in UK Biobank for categories of LTC counts and MLTC clusters. N represents number of participants per group in the accompanying regression model. Models included either LTC count categories as predictor (**A**, **C**) or MLTC clusters as predictor (**B**, **D**). Adjusted predictions calculated using the margins command in Stata with all covariates held constant at their mean values. Covariates: age, sex, ethnicity, deprivation (Townsend quintile), BMI, smoking, alcohol frequency, alcohol weekly units, physical activity, frailty phenotype, and baseline self-rated health.

**Fig. 2:**
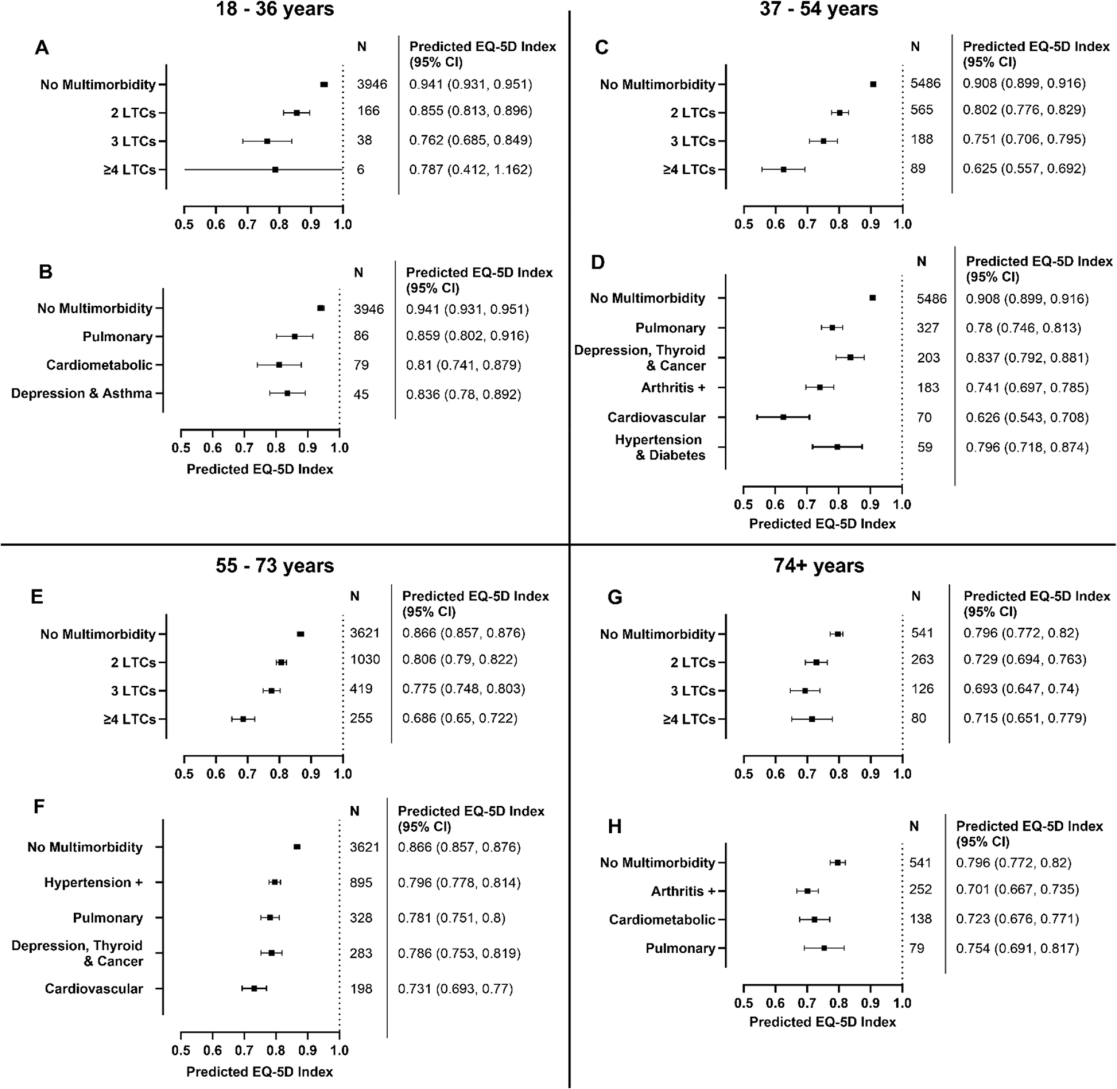
Predicted values for EQ-5D index at 5-year follow-up in UKHLS for categories of LTC counts and MLTC clusters. N represents number of participants per group in the accompanying regression model. Models included either LTC count categories as predictor (**A** , **C**, **E**, **G**) or MLTC clusters as predictor (**B**, **D**, **F**, **H**). Adjusted predictions calculated using the margins command in Stata with all covariates held constant at their mean values. Covariates: age, sex, ethnicity, deprivation (IMD quintile), BMI, smoking, alcohol frequency, physical activity, and baseline EQ index.

Adjusted predictions for EQ-5D index by LTC counts and MLTC clusters are presented in Figs. 1 and 2 for UK Biobank and UKHLS, respectively.

In UK Biobank, the adjusted predictions for EQ-5D index values at approx. 10-year follow-up were lowest in the ≥ 4 LTCs groups when compared to other LTC counts or MLTC clusters in the 37–54 years (0.777 [95%CI 0.765, 0.79]) and 55–73 years (0.796 [95%CI 0.79, 0.802]) groups (Fig. 1A & B). In the 37–54 years group, predicted value for the ‘*Depression and anxiety*’ cluster (0.795 [95%CI 0.784, 0.805]) and ‘*Pain* + ’ cluster (0.804 [95%CI 0.795, 0.814]) were the lowest of the MLTC clusters (Fig. 1C). In the 55–73 years group, ‘*Pain* + ’ had the poorest predicted EQ-5D index value (0.815 [95%CI 0.811, 0.819]) (Fig. 1D).

In UKHLS, adjusted predictions for EQ-5D index values at approx. 5-year follow-up differed by age group. ‘*Cardiovascular*’ clusters in the two middle age groups had the lowest predicted EQ-5D index values of all MLTC clusters in this cohort (0.626 [95%CI 0.543, 0.708], Fig. 2D), and 0.731 [95%CI 0.693, 0.77], Fig. 2F). Predicted EQ-5D index value for the ‘*Cardiovascular*’ cluster in the 37–54 years age strata was similar to the predicted value for having ≥ 4 LTCs (0.625 [95%CI 0.557, 0.692], Fig. 2C).

## Discussion

This study uniquely addresses the relationship between MLTC clusters and LTC counts on longitudinal HRQoL and compares findings across two large national cohort data sets. In this study, we identified different MLTC clusters across the adult age span and highlighted how the negative associations between MLTCs and HRQoL differed between clusters. Elucidating the associations between MLTC clusters (such as chronic pain and cardiometabolic clusters) and HRQoL may improve understanding of underlying mechanisms, identify targets for future interventions, and inform intervention design to enhance the likelihood of improving HRQoL in specific subgroups of people with MLTCs. MLTC clusters with conditions like chronic pain, depression, and cardiovascular disease were routinely associated with large deficits in HRQoL. People with such MLTC clusters may benefit from development of more targeted interventions for improvement of outcomes. We report negative associations between increasing LTC numbers and HRQoL but suggest that MLTC clusters may be equally important predictors of HRQoL across some ages. Notably, the negative association between MLTC clusters with chronic pain and HRQoL remained significant even after adjusting for number of LTCs.

Identifying MLTC clusters and their associated burden has been identified as a key priority to inform planning and development of effective healthcare services, potential treatments, and interventions, and to advance studies of mechanisms of MLTCs [1, 13]. Replication of MLTC clusters has been difficult and utilising different methodologies has been found to produce some divergent results even in the same data [17]. A MLTC cluster containing pulmonary conditions was consistently found across different age groups in our analyses, primarily driven by high prevalence of asthma in younger ages with increasing COPD prevalence in older groups. This is consistent with a recent systematic review of MLTC clustering methods, in which clusters of pulmonary conditions characterised by these two LTCs were one of three cluster types that were systematically identified when using LCA [19]. Clusters of cardiometabolic conditions and mental health disorders were also commonplace in MLTC clustering studies, regardless of the method used [19, 38]—again in support of the clusters identified in our analyses. The inverse association between number of co-existing LTCs and HRQoL has been previously reported [22] and is validated by our analyses. The negative association between numbers of LTCs and HRQoL were stronger in the UKHLS compared to UK Biobank cohort across comparable age groups (37–54 years & 55–73 years). For example, predicted HRQoL in the ≥ 4 LTCs group in UK Biobank was similar (0.796 [95%CI 0.790, 0.802]) to predicted HRQoL for the 2 LTC group in UKHLS (0.802 [95%CI 0.775, 0.829]) in the 37–54 years age group. These data suggest the impact of MLTCs on HRQoL were more pronounced in UKHLS, potentially owing to differences in sociodemographic or other unmeasured potential confounders. We acknowledge, however, that direct comparisons between these two cohorts are limited by differences in the LTCs considered, mapping of HRQoL data, and follow-up time, and suggest that future work assessing the role of more concordant assessments of MLTCs and HRQoL across diverse populations is needed.

Currently, investigations of longitudinal associations between MLTC clusters and long-term HRQoL are lacking. In cross-sectional studies, combined physical-mental MLTCs were associated with poorest self-rated heath in older European adults [39]. On the other hand, complex cardiometabolic and complex respiratory MLTC clusters, identified via LCA, had the poorest HRQoL in a large, Danish population-based study, scoring around 10–20% lower on both physical and mental components of the SF-12 questionnaire compared to the ‘relatively healthy’ group [16]. Whether these associations were driven by the specific cluster of LTCs or the number of co-existing conditions was unclear as number of conditions was not accounted for in the analyses. In our analyses, MLTC clusters centred around high prevalence of cardiovascular and metabolic diseases were also observed to have the largest deficits in HRQoL in the UKHLS cohort, particularly in the middle-aged groups. These associations were attenuated after adjustment for number of conditions, we expect due to underpowered analyses, and validation and analyses of HRQoL in larger clusters with similar morbidity profiles would be beneficial. These clusters, interestingly, contained the highest proportions of men and people from the most socioeconomically deprived areas, who may be at increased risk from unhealthy lifestyle factors and thus require complex interventions with consideration of sociodemographic factors [40].

In the above-mentioned Danish study, mental health and musculoskeletal clusters were also associated with significantly lower HRQoL compared to the healthy group [16], in accordance with our observations in UK Biobank of the largest deficits in HRQoL occurring in clusters centred around painful conditions and depression. These were also the only clusters that remained negatively associated with HRQoL after adjustment for LTC count. These data are further supported by another UK study of eleven single condition LTCs, which reported chronic pain and depression to be associated with the greatest deficits in cross-sectional EQ-5D index scores [41]. Painful conditions (including arthritis in UKHLS) and depression were prominent across many of the identified MLTC clusters in this study and others [7], and may represent potential targets for interventions which aim to improve HRQoL in people living with MLTCs.

### Implications

Improving HRQoL is a critical outcome for people living with MLTCs and their healthcare providers [20, 21]. Here, we have shown that the burden of reduced HRQoL in MLTCs is dependent not only on the number but also the cluster of MLTCs that are present. Both factors should be considered in the design of future interventions aimed at increasing HRQoL in populations with MLTCs, while further investigation of common mechanisms underpinning reduced HRQoL in specific MLTC clusters is also required. Disappointingly, previous interventions targeting people with MLTCs, evaluated using randomised clinical trials and designed to improve patient outcomes such as HRQoL, have been largely ineffective. A pooled analysis of interventions that used a variety of methodological approaches, including organisational change, promotion of self-management and medication management strategies, reported no evidence of benefit to mental health or HRQoL outcomes (including EQ-5D) [42]. Interventions typically included older adults with MLTCs, who according to our and others’ data may experience lesser detriment to HRQoL compared to younger populations [43], and despite the greatest burden of MLTCs being in those aged < 65 years [5, 6]. However, when studies have been designed to address specific comorbidities, such as depression, there have been reported improvements in depressive symptoms [5, 6]. Most interventions adopt a uniform approach to MLTC by using a simple count-based criteria, assuming that the MLTC population is, to a large extent, homogenous. Our data suggests such approaches may be ineffective due to populations with different morbidity profiles having divergent needs, including differences in long-term HRQoL. Accounting for these different needs in service and intervention design may increase the effectiveness of interventions aiming to improve HRQoL and consideration of MLTC clusters and counts should be implemented at this stage in future.

Hypertension had the highest single condition LTC prevalence in both cohorts and appeared as one of the top three most prevalent LTCs in 13/15 clusters in UKHLS and 6/9 clusters in UK Biobank, further highlighting its pervasiveness among people with MLTCs [7, 16, 44]. Similarly, painful conditions, depression, and arthritis were prevalent in many of the observed MLTC clusters. There is an urgent need to ensure interventions address the increased complexity of managing these issues in the context of MLTCs. Additionally, health services and research programmes for people with MLTCs should take greater account of the specific needs of people, depending on types of MLTC clusters, when designing services or interventions. Our ongoing NIHR funded research programme, PERFORM (NIHR202020), is testing an integrated service of personalised exercise rehabilitation and self-management strategies for people living with MLTCs, taking account of our findings regarding MLTC clusters and HRQoL when designing the intervention and trial eligibility criteria [45].

### Limitations

The measurement of MLTCs differed across the two datasets due to different data collection methods and both studies relied on self-reported data on LTCs which may be prone to recall bias. The prevalence of MLTCs in UKHLS (~ 18%) was likely underestimated due to the small number of LTCs used in its measurement (*n* = 13). A major challenge of MLTC research has been the lack of consensus in definitions and methodological approaches, which can lead to highly variable estimates of MLTC prevalence [46, 47]. Future MLTC research could benefit from cohorts, clinical trials, and other datasets implementing a common core set of LTCs to define MLTCs, in line with recent recommendations [47, 48]. This will enhance comparability across research studies and facilitate improved understanding of which combinations of MLTCs share common mechanisms or have the greatest impact on health outcomes and healthcare utilisation. Furthermore, a core MLTC definition should improve operationalisation into clinical practice and may better capture the clinical heterogeneity of MLTCs in comparison to previously developed weighted comorbidity indexes.

This study did not account for changes in MLTC over time and we used a static measure of MLTC at baseline. The MLTC profile of many individuals in our analyses may have changed over time and the acute effects of newly diagnosed LTCs could impact on HRQoL, including in people who reported no LTCs at baseline. Future work determining how MLTC clusters transition over time and how this affects the trajectory of HRQoL, healthcare costs, and health outcomes is urgently required.

Previous research has highlighted the role of social and behavioural determinants and how these factors would influence the onset and progression of MLTCs. Our objective was to compare the pattern of MLTCs and relationship with HRQOL across the two cohorts. We did not utilise social and behavioural factors while classifying MLTCs using LCA. This is an important limitation of our findings. However, Nguyen et al. applied LCA for MLTCs classification without and with inclusion of social and behavioural factors to find consistent MLTC clusters with both these approaches, respectively [49]. As a sensitivity analysis to test the influence of SES on MLTC clusters, we repeated LCA including SES as an indicator variable (similar to the approach of Nguyen et al.). The composition of MLTC clusters remained broadly similar in UK Biobank and almost completely unchanged in UKHLS when SES was included in the LCA models.

The UKHLS baseline cohort was broadly representative of 2011 census data from the UK Office for National Statistics (ONS), with the exception of some under-representation of men, people with severely limiting long-term illness, and people from Greater London. However, attrition rates were socially patterned, with higher attrition in people who were younger, from minority ethnic backgrounds, on lower incomes, and male [50, 51]. In contrast, the UK Biobank cohort is known to be healthier than the general adult population, with lower rates of smoking, morbidity and mortality, and being less likely to live in socioeconomically deprived areas [52]. Comparison of participants with and without follow-up data (Additional File 1: Table S10) also highlighted socially patterned attrition in UK Biobank, and this should be considered when evaluating our results. Both cohorts suffered from high levels of attrition and loss to follow-up was greater than 50% (> 75% in the oldest age group in UKHLS), which reduced the statistical power of our analyses, particularly in UKHLS, due to the small numbers of participants in regression models. Nevertheless, our analyses show the importance of MLTC clusters on long-term HRQoL across both cohorts. As our analyses were biased towards participants with generally better health status at baseline in each cohort, our estimated associations between MLTCs and HRQoL are likely to be conservative. Further work is needed to improve the collection and analysis of HRQoL data in routine healthcare settings and more representative cohort studies.

Our analyses investigated HRQoL at different follow-up periods in each cohort. Time may be an important determinant of HRQoL for people with MLTCs, particularly in groups with high risk of mortality. The higher rate of mortality from baseline in MLTC clusters compared to the ‘no multimorbidity’ group in UK Biobank, and HRQoL questionnaire being completed by only a sub-group of people, confounds follow-up, as data were available for a smaller proportion of participants in the MLTC clusters. This is perhaps reflected in our UK Biobank results, where the adverse associations between MLTC and HRQoL are smaller in the older age group, potentially owing to survival and selection bias.

We assigned individuals to MLTC clusters based on the highest predicted probability of cluster membership, an approach that has been commonly applied in prior MLTC research [7, 53–55]. In both simulated and healthcare data, this method was found to outperform other clustering algorithms in correctly assigning individuals to known MLTC clusters [17], and the mean predicted probabilities of cluster membership in our analyses were > 0.7 in all but one case (Table 2), indicating good distinction between clusters [56]. Nevertheless, this method can introduce the possibility of classification error and may bias estimates with distal outcomes (in this case, HRQoL) towards the null, and methods to mitigate this have been proposed [57]. Another potential limitation of LCA is subjectivity in model selection, as no consensus exists for a single ‘best’ indicator of the optimal model [34]. For transparency, a detailed explanation of our LCA methods and model selection process has been published online [12]. Additionally, as LCA is a prevalence-based approach, LTCs with low cohort prevalence do not feature prominently in identified clusters. It is, therefore, possible that smaller but potentially impactful MLTC clusters were unrecognised in our analyses and alternative approaches may be required to elucidate these important subgroups [58].

Finally, we mapped EQ-5D-5L responses onto the EQ-5D-3L UK value set using crosswalk functions as previously suggested by NICE [29]. Others have advocated for the use of mixture models [59], while a multi-equation mapping framework was recently developed by Alava et al. [60]. While this framework was found to perform better than crosswalk in translating EQ-5D-5L to EQ-5D-3L, there were no larger differences between the two methods and we therefore would not expect large deviation in findings had we adopted this approach.

### Conclusions

We have identified an association between MLTC clusters and long-term poor HRQoL irrespective of LTC count. Chronic pain and cardiometabolic MLTC clusters had a larger negative impact than other clusters. This has important clinical implications for risk assessment and clinical management of people with these MLTC clusters. Raising awareness of these issues is important for medical educators and policy makers and to inform and influence service design and lead to better support for these patients. These findings may also influence future work in MLTC trials, when designing and developing interventions to address common underlying symptoms or mechanisms and when considering inclusion criteria for MLTC intervention trials, especially where HRQoL is a key outcome measure.

## Supplementary Information

Additional file 1. Table S1. List of self-reported long-term conditions used in each cohort for assessment of MLTC. Table S2. Prevalence of self-reported LTCs at baseline in UK Biobank and UKHLS. Table S3. Cohort characteristics by MLTC clusters in UK Biobank participants aged 37 – 54 years. Table S4. Cohort characteristics by MLTC clusters in UK Biobank participants aged 55 - 73 years. Table S5. Cohort characteristics by MLTC clusters in UKHLS participants aged 18 – 36 years. Table S6. Cohort characteristics by MLTC clusters in UKHLS participants aged 37 – 54 years. Table S7. Cohort characteristics by MLTC clusters in UKHLS participants aged 55 – 73 years. Table S8. Cohort characteristics by MLTC clusters in UKHLS participants aged 74+ years. Table S9. Comparison of baseline cohort characteristics in participants with and without HRQoL follow up data in UK Biobank. Table S10. Comparison of baseline cohort characteristics in participants with and without HRQoL follow up data in UKHLS. Table S11.Sensitivity analysis: Summary of MLTC clusters when socioeconomic status included as a variable in LCA models. Table S12. Association between MLTC and HRQoL at 10-years follow up in UK Biobank: combined model with MLTC clusters and LTC counts. Table S13. Association between MLTC and HRQoL at 5-years follow up in UKHLS: combined model with MLTC clusters and LTC counts. Table S14. Sensitivity analyses: OLS regression models for EQ-5D Index at 10-years follow up in UK Biobank. Table S15. Sensitivity analyses: OLS regression results for EQ-5D Index scores at median 5-year follow up in UKHLS. Figure S1. Flow diagram of participant inclusion in UK Biobank analyses. Figure S2. Flow diagram of participant inclusion in UKHLS analyses.

## Data Availability

The data that support these findings are available from UK Biobank and the UK Data service, subject to successful registration and application processes. Access to data from UK Biobank can be requested via the UK Biobank Access Management System https://www.ukbiobank.ac.uk/. Access to UKHLS data can be requested via UK Data Service https://ukdataservice.ac.uk/.

## Abbreviations

aBIC: Sample size adjusted Bayesian information criterion
BIC: Bayesian information criterion
COPD: Chronic obstructive pulmonary disease
EQ-5D: EuroQol five dimension quality of life questionnaire
HRQoL: Health-related quality of life
IMD: Index of multiple deprivation
IQR: Inter-quartile range
LCA: Latent class analysis
LTC: Long-term condition
MLTC(s): Multiple long-term conditions
UKHLS: UK Household Longitudinal Study
