# Supplementary material for: Multimorbidity clusters and their associations with health-related quality of life in two UK cohorts
Source: BMC Med. 2025 Jan 8;23:1. doi: 10.1186/s12916-024-03811-3 (PMC11708164; doi:10.1186/s12916-024-03811-3)
Supplement: Supplementary file 1 — Additional file 1. Table S1. List of self-reported long-term conditions used in each cohort for assessment of MLTC. Table S2. Prevalence of self-reported LTCs at baseline in UK Biobank and UKHLS. Table S3. Cohort characteristics by MLTC clusters in UK Biobank participants aged 37 – 54 years. Table S4. Cohort characteristics by MLTC clusters in UK Biobank participants aged 55 - 73 years. Table S5. Cohort characteristics by MLTC clusters in UKHLS participants aged 18 – 36 years. Table S6. Cohort characteristics by MLTC clusters in UKHLS participants aged 37 – 54 years. Table S7. Cohort characteristics by MLTC clusters in UKHLS participants aged 55 – 73 years. Table S8. Cohort characteristics by MLTC clusters in UKHLS participants aged 74+ years. Table S9. Comparison of baseline cohort characteristics in participants with and without HRQoL follow up data in UK Biobank. Table S10. Comparison of baseline cohort characteristics in participants with and without HRQoL follow up data in UKHLS. Table S11.Sensitivity analysis: Summary of MLTC clusters when socioeconomic status included as a variable in LCA models. Table S12. Association between MLTC and HRQoL at 10-years follow up in UK Biobank: combined model with MLTC clusters and LTC counts. Table S13. Association between MLTC and HRQoL at 5-years follow up in UKHLS: combined model with MLTC clusters and LTC counts. Table S14. Sensitivity analyses: OLS regression models for EQ-5D Index at 10-years follow up in UK Biobank. Table S15. Sensitivity analyses: OLS regression results for EQ-5D Index scores at median 5-year follow up in UKHLS. Figure S1. Flow diagram of participant inclusion in UK Biobank analyses. Figure S2. Flow diagram of participant inclusion in UKHLS analyses. [file 12916_2024_3811_MOESM1_ESM.docx]

# Additional Material

### Tables

| Table S1. | List of self-reported long-term conditions used in each cohort for assessment of MLTC. |
| --- | --- |
| Table S2. | Prevalence of self-reported LTCs at baseline in UK Biobank and UKHLS. |
| Table S3. | Cohort characteristics by MLTC clusters in UK Biobank participants aged 37 – 54 years. |
| Table S4. | Cohort characteristics by MLTC clusters in UK Biobank participants aged 55 - 73 years. |
| Table S5. | Cohort characteristics by MLTC clusters in UKHLS participants aged 18 – 36 years. |
| Table S6. | Cohort characteristics by MLTC clusters in UKHLS participants aged 37 – 54 years. |
| Table S7. | Cohort characteristics by MLTC clusters in UKHLS participants aged 55 – 73 years. |
| Table S8. | Cohort characteristics by MLTC clusters in UKHLS participants aged 74+ years. |
| Table S9. | Comparison of baseline cohort characteristics in participants with and without HRQoL follow up data in UK Biobank |
| Table S10. | Comparison of baseline cohort characteristics in participants with and without HRQoL follow up data in UKHLS. |
| Table S11. | Sensitivity analysis: Summary of MLTC clusters when socioeconomic status included as a variable in LCA models. |
| Table S12. | Association between MLTC and HRQoL at 10-years follow up in UK Biobank: combined model with MLTC clusters and LTC counts. |
| Table S13. | Association between MLTC and HRQoL at 5-years follow up in UKHLS: combined model with MLTC clusters and LTC counts. |
| Table S14. | Sensitivity analyses: OLS regression models for EQ-5D Index at 10-years follow up in UK Biobank. |
| Table S15. | Sensitivity analyses: OLS regression results for EQ-5D Index scores at median 5-year follow up in UKHLS. |

### Figures

| Figure S1. | Flow diagram of participant inclusion in UK Biobank analyses. |
| --- | --- |
| Figure S2. | Flow diagram of participant inclusion in UKHLS analyses. |

Table S1. List of self-reported long-term conditions used in each cohort for assessment of MLTC.

| ***Long term conditions*** | ***Conditions included in definition from self-report (UK Biobank)*** | ***Conditions included in definition from self-report (UKHLS)*** |
| --- | --- | --- |
| **Hypertension** | Hypertension  Essential hypertension | Hypertension |
| Painful conditions | Back pain  Joint pain  Back pain  Joint pain  Headaches (not migraine)  Sciatica  Plantar fasciitis  Carpal tunnel syndrome  Fibromyalgia  Arthritis  Shingles  Disc problem  Prolapsed disc/slipped disc  Spine arthritis/spondylitis  Ankylosing spondylitis  Back problem  Osteoarthritis  Gout  Cervical spondylosis  Trigeminal neuralgia  Disc degeneration  Trapped nerve/compressed nerve |  |
| **Asthma** | Asthma | Asthma |
| Dyspepsia | Gastro-oesophageal reflux/gastric reflux  Oesophagitis /Barrett's oesophagus  Gastric stomach ulcers  Gastric erosions/gastritis  Duodenal ulcer  Dyspepsia/indigestion  Hiatus hernia  Helicobacter pylori |  |
| **Cancer** | Any lifetime diagnosis of cancer | Cancer or malignancy |
| **Thyroid disease** | Thyroid problem (not cancer)  Hyperthyroidism/thyrotoxicosis  Hypothyroidism/myxoedema  Grave’s disease  Thyroid goitre  Thyroiditis | Hyperthyroidism  Hypothyroidism |
| **Depression** | Depression  Postnatal depression | Depression |
| **Diabetes** | Diabetic nephropathy  Diabetic neuropathy/ulcers  Diabetes  Type 1 diabetes  Type 2 diabetes  Diabetic eye disease | Diabetes |
| **Coronary heart disease** | Heart attack/myocardial infarction  Angina | Coronary heart disease  Heart attack or myocardial infarction  Angina |
| Psoriasis or eczema | Eczema  Dermatitis  Psoriasis |  |
| Migraine | Migraine |  |
| Irritable bowel syndrome | Irritable bowel syndrome |  |
| Rheumatoid arthritis, connective tissue disease or other inflammatory arthropathies | Myositis/myopathy  Systemic Lupus Erythematosus  Connective tissue disorder  Sjogrens syndrome/sicca syndrome  Dermatomyositis  polymyositis  Scleroderma/systemic sclerosis  Rheumatoid arthritis  Psoriatic arthropathy  Dermatomyositis  Polymyositis  Polymyalgia Rheumatica  Malabsorption/coeliac disease |  |
| Anxiety | Anxiety/panic attacks  Nervous breakdown  Post-traumatic stress disorder  Obsessive compulsive disorder  Stress  Insomnia  Psychological/psychiatric problem |  |
| Stroke or TIA | Stroke  TIA  Subarachnoid haemorrhage  Brain haemorrhage  Ischaemic stroke | Stroke |
| **Chronic obstructive pulmonary disease (COPD)** | COPD/chronic obstructive airways disease  Emphysema/chronic bronchitis  Emphysema | Chronic bronchitis  Emphysema |
| Prostate disorders | Prostate problem (not cancer)  Enlarged prostate  Benign prostatic hypertrophy |  |
| Osteoporosis | Osteoporosis |  |
| Diverticular disease | Diverticular disease  Diverticulitis |  |
| Glaucoma | Glaucoma |  |
| Inflammatory bowel disease | Inflammatory bowel disease  Crohn’s disease  Ulcerative colitis |  |
| Endometriosis | Endometriosis |  |
| **Epilepsy** | Epilepsy | Epilepsy |
| Atrial Fibrillation | Atrial Fibrillation |  |
| Chronic sinusitis | Chronic sinusitis |  |
| Chronic fatigue syndrome | Chronic fatigue syndrome |  |
| Schizophrenia or bipolar disorders | Schizophrenia  Mania/bipolar disorder  Manic depression |  |
| Multiple sclerosis | Multiple sclerosis |  |
| Pernicious anaemia | Pernicious anaemia |  |
| Meniere's disease | Meniere's disease |  |
| Viral hepatitis | Infective/viral hepatitis  Hepatitis B  Hepatitis C  Hepatitis D  Hepatitis E |  |
| Chronic kidney disease | Polycystic kidney  Diabetic nephropathy  Renal/kidney failure  Renal failure requiring dialysis  Renal failure not requiring dialysis  Kidney nephropathy  Immunoglobulin A (IgA) nephropathy |  |
| Peripheral vascular disease | Peripheral vascular disease  Leg claudication/intermittent claudication |  |
| Bronchiectasis | Bronchiectasis |  |
| **Chronic liver disease** | Oesophageal varices  Non-infective hepatitis  Liver failure/cirrhosis  Primary biliary cirrhosis | Any Liver disease |
| Parkinson's disease | Parkinson's disease |  |
| Alcohol problems | Alcohol dependency  Alcoholic liver disease/alcoholic cirrhosis |  |
| **Heart failure** | Cardiomyopathy  Hypertrophic cardiomyopathy  Heart failure/pulmonary oedema | Heart failure |
| Polycystic ovarian syndrome | Polycystic ovarian syndrome |  |
| Treated constipation | Constipation |  |
| Anorexia or bulimia | Anorexia  Bulimia  Other eating disorders |  |
| Dementia | Dementia  Alzheimer’s disease  Cognitive impairment |  |
| Psychoactive substance misuse | Opioid dependency  Other substance abuse/dependency |  |
| Arthritis |  | Arthritis |

**Bolded** LTCs appear in both datasets (n=12).

Table S2. Prevalence of self-reported LTCs at baseline in UK Biobank and UKHLS.

| **UK BIOBANK (n = 502,363)** | |  | **UKHLS (n = 49,186)** | |
| --- | --- | --- | --- | --- |
| **LTC (n = 43)** | **N (%)** |  | **LTC (n = 13)** | **N (%)** |
| **Hypertension** | 133268 (26.5) |  | **Hypertension** | 8690 (17.7) |
| Painful conditions | 83945 (16.7) |  | Arthritis | 6767 (13.8) |
| **Asthma** | 58258 (11.6) |  | **Asthma** | 5973 (12.1) |
| Dyspepsia | 39059 (7.8) |  | **Depression** | 3155 (6.4) |
| **Cancer** | 38608 (7.7) |  | **Diabetes** | 2865 (5.8) |
| **Thyroid disease** | 29121 (5.8) |  | **Coronary heart disease** | 2413 (4.9) |
| **Depression** | 28459 (5.7) |  | **Thyroid disease** | 1729 (3.5) |
| **Diabetes** | 25492 (5.1) |  | **Cancer** | 1622 (3.3) |
| **Coronary heart disease** | 22720 (4.5) |  | **COPD** | 1187 (2.4) |
| Psoriasis or eczema | 17827 (3.6) |  | **Stroke** | 864 (1.8) |
| Migraine | 14380 (2.9) |  | **Liver disease** | 586 (1.2) |
| Irritable bowel syndrome | 11487 (2.3) |  | **Epilepsy** | 511 (1.0) |
| Rheumatoid arthritis, connective tissue disease or other inflammatory arthropathies | 11026 (2.2) |  | **Heart failure** | 276 (0.6) |
| Anxiety | 9024 (1.8) |  |  |  |
| **Stroke or TIA** | 8851 (1.8) |  |  |  |
| **COPD** | 8312 (1.7) |  |  |  |
| Prostate disorders | 8256 (1.6) |  |  |  |
| Osteoporosis | 8039 (1.6) |  |  |  |
| Diverticular disease | 5401 (1.1) |  |  |  |
| Glaucoma | 5313 (1.1) |  |  |  |
| Inflammatory bowel disease | 4231 (0.8) |  |  |  |
| Endometriosis | 4056 (0.8) |  |  |  |
| **Epilepsy** | 4051 (0.8) |  |  |  |
| Atrial Fibrillation | 3650 (0.7) |  |  |  |
| Chronic sinusitis | 3102 (0.6) |  |  |  |
| Chronic fatigue syndrome | 2166 (0.4) |  |  |  |
| Schizophrenia or bipolar disorders | 1995 (0.4) |  |  |  |
| Multiple sclerosis | 1777 (0.4) |  |  |  |
| Pernicious anaemia | 1517 (0.3) |  |  |  |
| Meniere's disease | 1376 (0.3) |  |  |  |
| Viral hepatitis | 1336 (0.3) |  |  |  |
| Chronic kidney disease | 1310 (0.3) |  |  |  |
| Peripheral vascular disease | 1278 (0.3) |  |  |  |
| Bronchiectasis | 1135 (0.2) |  |  |  |
| **Chronic liver disease** | 969 (0.2) |  |  |  |
| Parkinson's disease | 857 (0.2) |  |  |  |
| Alcohol problems | 808 (0.2) |  |  |  |
| **Heart failure** | 803 (0.2) |  |  |  |
| Polycystic ovarian syndrome | 622 (0.1) |  |  |  |
| Treated constipation | 403 (0.1) |  |  |  |
| Anorexia or bulimia | 370 (0.1) |  |  |  |
| Dementia | 124 (0.0) |  |  |  |
| Psychoactive substance misuse | 98 (0.0) |  |  |  |

**Bolded** LTCs appear in both datasets (n=12).

Table S3. Cohort characteristics by MLTC clusters in UK Biobank participants aged 37 – 54 years.

|  | **No multimorbidity** | **Hypertension & Diabetes** | **Asthma +** | **Pain +** | **Depression & Anxiety** | **Cancer & Thyroid** |
| --- | --- | --- | --- | --- | --- | --- |
| N (%) | 152,975 (78.3) | 16,126 (8.3) | 9680 (5.0) | 5072 (2.6) | 5250 (2.7) | 6006 (3.1) |
| Female sex, n (%) | 82,995 (54.6) | 7595 (47.1) | 6175 (63.8) | 3257 (64.2) | 3705 (70.5) | 4205 (70) |
| Age (years), median (IQR) | 48 (44, 51) | 50 (46, 53) | 48 (44, 51) | 49 (45, 52) | 48 (44, 52) | 49 (45, 52) |
| White ethnicity, n (%) | 138,121 (91.4) | 14,279 (89.1) | 8958 (93.0) | 4685 (92.8) | 4945 (94.7) | 5580 (93.4) |
| Number of LTCs, median (IQR) | 0 (0, 1) | 2 (2, 3) | 2 (2, 3) | 2 (2, 3) | 2 (2, 3) | 2 (2, 2) |
| Socioeconomic deprivation ^a^, n (%) |  |  |  |  |  |  |
| 1 (Most affluent) | 60,622 (39.9) | 5011 (31.1) | 3438 (35.5) | 1726 (34.0) | 1557 (29.7) | 2153 (35.9) |
| 2 | 19,727 (13.0) | 1874 (11.6) | 1184 (12.2) | 638 (12.6) | 656 (12.5) | 731 (12.2) |
| 3 | 20,592 (13.6) | 2067 (12.8) | 1225 (12.7) | 682 (13.5) | 650 (12.4) | 788 (13.1) |
| 4 | 25,201 (16.6) | 3057 (19.0) | 1747 (18.1) | 905 (17.8) | 954 (18.2) | 1047 (17.4) |
| 5 (Most deprived) | 25,587 (16.8) | 4084 (25.3) | 2065 (21.3) | 1114 (22.0) | 1418 (27.0) | 1279 (21.3) |
| Missing | 246 (0.2) | 33 (0.2) | 21 (0.2) | 7 (0.1) | 15 (0.3) | 8 (0.1) |
| BMI (kg/m^2^) | 26.0 (23.5, 29.1) | 29.9 (26.6, 34.1) | 27.0 (24.1, 30.7) | 27.4 (24.3, 30.9) | 27.3 (24.1, 31.4) | 26.2 (23.4, 29.7) |
| Alcohol intake (weekly units), median (IQR) | 9.5 (1.4, 21) | 9 (0, 24) | 7 (0, 18) | 6 (0, 18) | 4.5 (0, 17) | 5.5 (0, 15) |
| Alcohol frequency, n (%) |  |  |  |  |  |  |
| Never | 10,102 (6.7) | 1809 (11.2) | 928 (9.6) | 567 (11.2) | 765 (14.6) | 735 (12.2) |
| Special occasions only | 15,158 (10.0) | 2364 (14.7) | 1413 (14.6) | 809 (16.0) | 874 (16.7) | 940 (15.7) |
| 1-3 times per month | 18,850 (12.4) | 2133 (13.2) | 1363 (14.1) | 758 (14.9) | 772 (14.7) | 887 (14.8) |
| 1-2 times per week | 44,054 (29.0) | 4025 (25.0) | 2522 (26.1) | 1297 (25.6) | 1194 (22.7) | 1547 (25.8) |
| 3-4 times per week | 37,734 (24.8) | 3093 (19.2) | 2026 (20.9) | 91 (18.0) | 849 (16.2) | 1126 (18.8) |
| Daily/almost daily | 25,540 (16.8) | 2651 (16.4) | 1403 (14.5) | 715 (14.1) | 761 (14.5) | 754 (12.6) |
| Missing | 537 (0.4) | 51 (0.3) | 25 (0.3) | 15 (0.3) | 35 (0.7) | 17 (0.3) |
| Smoking, n (%) |  |  |  |  |  |  |
| Never | 93,293 (61.4) | 8783 (54.4) | 5481 (56.6) | 2753 (54.3) | 2675 (50.9) | 3367 (56.1) |
| Previous | 39,106 (25.7) | 4792 (29.7) | 2684 (27.7) | 1432 (28.2) | 1392 (26.5) | 1717 (28.6) |
| Current | 18,802 (12.4) | 2475 (15.4) | 1465 (15.1) | 854 (16.8) | 1161 (22.1) | 894 (14.9) |
| Missing | 774 (0.5) | 76 (0.5) | 50 (0.5) | 33 (0.7) | 22 (0.4) | 28 (0.5) |
| Physical activity, n (%) |  |  |  |  |  |  |
| High | 25,984 (17.1) | 1256 (7.8) | 1175 (12.1) | 446 (8.8) | 355 (6.8) | 544 (9.1) |
| Moderate | 110,615 (72.7) | 11,697 (72.5) | 7113 (73.5) | 3726 (73.5) | 3799 (72.4) | 4570 (76.1) |
| Low | 4390 (2.9) | 793 (4.9) | 385 (4.0) | 237 (4.7) | 281 (5.4) | 237 (4.0) |
| None | 8725 (5.7) | 2101 (13.0) | 894 (9.2) | 601 (11.9) | 743 (14.2) | 575 (9.6) |
| Missing | 2261 (1.5) | 279 (1.7) | 113 (1.2) | 62 (1.2) | 72 (1.4) | 80 (1.3) |
| Frailty phenotype ^b^, n (%) |  |  |  |  |  |  |
| Robust | 96,507 (63.5) | 6511 (40.4) | 4713 (48.7) | 2310 (45.5) | 1812 (34.5) | 2904 (48.4) |
| Pre-frail | 50,005 (32.9) | 7757 (48.1) | 4183 (43.2) | 2195 (43.3) | 2720 (51.8) | 2652 (44.2) |
| Frail | 2128 (1.4) | 1372 (8.5) | 589 (6.1) | 448 (8.8) | 584 (11.1) | 314 (5.2) |
| Missing | 3335 (2.2) | 486 (3.0) | 195 (2.0) | 119 (2.4) | 134 (2.6) | 136 (2.3) |
| Self-rated health, n (%) |  |  |  |  |  |  |
| Excellent | 32,014 (21.1) | 608 (3.8) | 748 (7.7) | 295 (5.8) | 239 (4.6) | 449 (7.5) |
| Good | 91,191 (60.0) | 6508 (40.4) | 4720 (48.8) | 2339 (46.1) | 1984 (37.8) | 2903 (48.3) |
| Fair | 24,641(16.2) | 6269 (38.9) | 3083 (31.9) | 1710 (33.7) | 1929 (36.7) | 1934 (32.2) |
| Poor | 3008 (2.0) | 2555 (15.8) | 1052 (10.9) | 678 (13.4) | 1023 (19.5) | 654 (10.9) |
| Missing | 1121 (0.7) | 186 (1.2) | 77 (0.8) | 50 (1.0) | 75 (1.4) | 66 (1.1) |

^a^ Using UK population distribution of Townsend deprivation scores from 2001 Census data. ^b^ Freid frailty phenotype.

Table S4. Cohort characteristics by MLTC clusters in UK Biobank participants aged 55 - 73 years.

|  | **No multimorbidity** | **Cancer +** | **Pain +** | **Cardiometabolic** | **Pulmonary** |
| --- | --- | --- | --- | --- | --- |
| N (%) | 185,265 (60.1) | 16,460 (5.3) | 33,165 (10.8) | 52,740 (17.1) | 20,624 (6.7) |
| Female sex, n (%) | 98,778 (53.3) | 10,259 (62.3) | 21,891 (66.0) | 21,982 (41.7) | 12,520 (60.7) |
| Age (years), median (IQR) | 61 (58, 65) | 63 (60, 66) | 62 (59, 66) | 63 (60, 66) | 62 (59, 65) |
| White ethnicity, n (%) | 178,325 (96.3) | 16,084 (97.7) | 32,227 (97.2) | 49,562 (94.0) | 19,803 (96.0) |
| Number of LTCs (from n = 43), median (IQR) | 1 (0, 1) | 2 (2, 3) | 2 (2, 3) | 2 (2, 3) | 2 (2, 3) |
| Socioeconomic deprivation ^a^, n (%) |  |  |  |  |  |
| 1 (Most affluent) | 87,638 (47.3) | 7415 (45.1) | 14,121 (42.6) | 20,625 (39.1) | 8782 (42.6) |
| 2 | 26,410 (14.3) | 2265 (13.8) | 4562 (13.8) | 6922 (13.1) | 2751 (13.3) |
| 3 | 23,962 (12.3) | 2055 (12.5) | 4338 (13.1) | 6791 (12.9) | 2666 (12.9) |
| 4 | 25,738 (13.9) | 2447 (14.9) | 4999 (15.1) | 8541 (16.2) | 3215 (15.6) |
| 5 (Most deprived) | 21,348 (11.5) | 2260 (13.7) | 5115 (15.4) | 9805 (18.6) | 3190 (15.5) |
| Missing | 169 (0.1) | 18 (0.1) | 30 (0.1) | 56 (0.1) | 20 (0.1) |
| BMI (kg/m^2^) | 26.3 (24.0, 29.1) | 27.1 (24.5, 30.3) | 27.1 (24.5, 30.3) | 29.2 (26.3, 32.7) | 27.5 (24.8, 30.1) |
| Alcohol intake (weekly units), median (IQR) | 9.5 (1.8, 21) | 7.5 (0, 18) | 6 (0, 15.5) | 9 (0, 22) | 7.5 (0, 18) |
| Alcohol frequency, n (%) |  |  |  |  |  |
| Never | 12,141 (6.6) | 1516 (9.2) | 3993 (12.0) | 5972 (11.3) | 2096 (10.2) |
| Special occasions only | 18,711 (10.1) | 2251 (13.7) | 5134 (15.5) | 7504 (14.2) | 2831 (13.7) |
| 1-3 times per month | 17,908 (9.7) | 1723 (10.5) | 3774 (11.4) | 5392 (10.2) | 2276 (11.0) |
| 1-2 times per week | 45,944 (24.8) | 3918 (23.8) | 7656 (23.1) | 12,342 (23.4) | 4758 (23.1) |
| 3-4 times per week | 45,216 (24.4) | 3400 (20.7) | 6313 (19.0) | 10,481 (19.9) | 4264 (20.7) |
| Daily/almost daily | 44,794 (24.2) | 3642 (22.1) | 6193 (18.7) | 10,930 (20.7) | 4361 (21.2) |
| Missing | 551 (0.3) | 10 (0.1) | 102 (0.3) | 119 (0.2) | 38 (0.2) |
| Smoking, n (%) |  |  |  |  |  |
| Never | 99,657 (53.8) | 7929 (48.2) | 16,207 (48.9) | 23,017 (43.6) | 10,286 (49.9) |
| Previous | 68,417 (36.9) | 7052 (42.8) | 13,358 (40.3) | 24,488 (46.4) | 8570 (41.6) |
| Current | 16,034 (8.7) | 1393 (8.5) | 3380 (10.2) | 4871 (9.2) | 1630 (7.9) |
| Missing | 1157 (0.6) | 86 (0.5) | 220 (0.7) | 364 (0.7) | 138 (0.7) |
| Physical activity, n (%) |  |  |  |  |  |
| High | 15,307 (8.3) | 767 (4.7) | 1309 (4.0) | 1879 (3.6) | 1033 (5.0) |
| Moderate | 153,650 (82.9) | 13,473 (81.9) | 26,800 (80.8) | 41,491 (78.8) | 16,455 (79.8) |
| Low | 5980 (3.2) | 786 (4.8) | 1786 (5.4) | 3026 (5.7) | 1032 (5.0) |
| None | 7875 (4.3) | 1238 (7.5) | 2855 (8.6) | 5415 (10.3) | 1815 (8.8) |
| Missing | 2453 (1.3) | 196 (1.2) | 415 (1.3) | 929 (1.8) | 289 (1.4) |
| Frailty phenotype ^b^, n (%) |  |  |  |  |  |
| Robust | 118,624 (64.0) | 8239 (50.1) | 15,144 (45.7) | 21,707 (41.2) | 9569 (46.4) |
| Pre-frail | 60,012 (32.4) | 6953 (42.2) | 14,790 (44.6) | 24,777 (47.0) | 9131 (44.3) |
| Frail | 2688 (1.5) | 923 (5.6) | 2422 (7.3) | 4675 (8.9) | 1419 (6.9) |
| Missing | 3941 (2.1) | 345 (2.1) | 809 (2.4) | 1518 (3.0) | 505 (2.5) |
| Self-rated health, n (%) |  |  |  |  |  |
| Excellent | 40,566 (21.9) | 1263 (7.7) | 2111 (6.4) | 2247 (4.3) | 1292 (6.3) |
| Good | 117,591 (63.5) | 8971 (54.5) | 17,237 (52.0) | 24,716 (46.8) | 10,786 (52.3) |
| Fair | 23,844 (12.9) | 4881 (29.7) | 10,690 (32.2) | 19,743 (37.4) | 6608 (32.0) |
| Poor | 2302 (1.2) | 1228 (7.5) | 2854 (8.6) | 5614 (10.6) | 1800 (8.7) |
| Missing | 962 (0.5) | 117 (0.7) | 273 (0.8) | 420 (0.8) | 138 (0.7) |

^a^ Using UK population distribution of Townsend deprivation scores from 2001 Census data. ^b^ Freid frailty phenotype.

Table S5. Cohort characteristics by MLTC clusters in UKHLS participants aged 18 – 36 years.

|  | **No multimorbidity** | **Cardiometabolic** | **Depression & Asthma** | **Pulmonary** |
| --- | --- | --- | --- | --- |
| N (%) | 15,416 (95.7) | 251 (1.6) | 168 (1.0) | 270 (1.7) |
| Female sex, n (%) | 8454 (54.8) | 175 (69.7) | 124 (73.8) | 176 (65.2) |
| Age (years), median (IQR) | 28 (23, 32) | 31 (27, 34) | 28 (24, 32) |  |
| White ethnicity, n (%) | 10,015 (68.3) | 213 (84.9) | 146 (86.9) | 226 (83.7) |
| Number of LTCs  (from n=13), median (IQR) | 0 (0, 0) | 2 (2, 3) | 2 (2, 3) | 2 (2, 3) |
| BMI (kg/m^2^), median (IQR) | 23.9 (21.4, 27.1) | 26.3 (22.7, 32.3) | 25.3 (21.3, 28.4) | 26.2 (22.3, 31.1) |
| Socioeconomic deprivation, n (%) |  |  |  |  |
| 1 (Most deprived) | 4560 (29.6) | 89 (35.5) | 65 (28.7) | 81 (30.0) |
| 2 | 3682 (23.9) | 66 (26.3) | 27 (16.1) | 62 (23.0) |
| 3 | 2861 (16.6) | 41 (16.3) | 30 (17.9) | 45 (16.7) |
| 4 | 2348 (15.2) | 33 (13.2) | 24 (14.3) | 46 (17.0) |
| 5 (Most affluent) | 1965 (12.8) | 22 (8.8) | 22 (13.3) | 36 (13.3) |
| Smoking *, n (%) |  |  |  |  |
| Never | 4483 (29.1) | 65 (25.9) | 29 (17.3) | 64 (23.7) |
| Previous | 2570 (16.7) | 38 (15.4) | 41 (24.4) | 52 (19.3) |
| Current | 2520 (16.4) | 73 (29.1) | 47 (28.0) | 67 (24.8) |
| Missing | 5843 (37.9) | 75 (29.9) | 51 (30.4) | 87 (32.2) |
| Alcohol *, n (%) |  |  |  |  |
| Never, or up to 2 times/year | 1929 (12.5) | 46 (18.3) | 27 (16.1) | 41 (15.2) |
| Up to 1-2 times/month | 2317 (15.0) | 50 (19.9) | 30 (17.9) | 51 (18.9) |
| 1-4 times/week | 3221 (20.9) | 40 (15.9) | 29 (17.3) | 56 (20.7) |
| Daily or almost daily | 430 (2.8) | 12 (4.8) | 7 (4.2) | 9 (3.3) |
| Missing | 7519 (48.8) | 103 (41.0) | 75 (44.6) | 113 (41.9) |
| Physical Activity score ^a^, median (IQR) | 5 (1, 16) | 3 (0, 12) | 6 (0, 14) | 4 (0, 14) |
| EQ-5D Index Score ^b^, median (IQR) | 1.00 (0.796, 1) | 0.727 (0.62, 0.848) | 0.727 (0.689, 0.848) | 0.796 (0.691, 1.00) |
| Self-rated health, n (%) |  |  |  |  |
| Excellent | 3947 (25.6) | 8 (3.2) | 9 (5.4) | 25 (9.3) |
| Very good | 5838 (37.9) | 47 (18.7) | 28 (16.7) | 56 (20.7) |
| Good | 4078 (26.5) | 60 (23.9) | 56 (33.3) | 84 (31.1) |
| Fair | 1212 (7.9) | 79 (31.5) | 45 (26.8) | 59 (21.9) |
| Poor | 313 (2.0) | 57 (22.7) | 30 (17.9) | 46 (17.0) |
| Missing | 28 (0.2) | 0 (0) | 0 (0) | 0 (0) |

* Data for smoking, alcohol intake and physical activity were not available at baseline and these data were collected at one-year follow up (Wave ‘b’ of UKHLS data collection). ^a^ Continuous count of number of days in past 4 weeks the participant had walked for at least 30 minutes (range 0 – 28). ^b^ EQ-5D index scores calculated from collected SF-12 data.

Table S6. Cohort characteristics by MLTC clusters in UKHLS participants aged 37 – 54 years.

|  | **No multimorbidity** | **Cardiovascular** | **Arthritis +** | **Pulmonary** | **Depression, Thyroid & Cancer** | **Diabetes & Hypertension** |
| --- | --- | --- | --- | --- | --- | --- |
| N (%) | 14,542 (86.9) | 218 (1.3) | 466 (2.8) | 805 (4.8) | 485 (2.9) | 210 (1.3) |
| Female sex, n (%) | 7758 (53.4) | 98 (45) | 313 (67.2) | 553 (68.7) | 315 (65) | 109 (51.9) |
| Age (years), median (IQR) | 45 (40, 49) | 49 (44, 52) | 49 (44, 52) | 46 (42, 51) | 47 (42, 51) | 49 (44, 52) |
| White ethnicity, n (%) | 11,054 (78.9) | 108 (51.4) | 693 (86.2) | 388 (83.3) | 174 (79.8) | 414 (85.4) |
| Number of LTCs  (from n=13), median (IQR) | 0 (0, 1) | 3 (2, 4) | 2 (2, 3) | 2 (2, 3) | 2 (2, 2) | 2 (2, 3) |
| BMI (kg/m^2^), median (IQR) | 25.8 (23.1, 29.0) | 28.7 (25.3, 32.0) | 28.1 (24.3, 32.9) | 27.3 (23.7, 32.0) | 28.1 (23.9, 33.0) | 30.8 (26.7, 35.5) |
| Socioeconomic deprivation, n (%) |  |  |  |  |  |  |
| 1 (Most deprived) | 3079 (21.2) | 92 (42.2) | 153 (32.8) | 242 (30.1) | 121 (25.0) | 94 (44.8) |
| 2 | 2886 (19.9) | 48 (22.0) | 102 (21.9) | 170 (21.1) | 104 (21.4) | 51 (24.3) |
| 3 | 2852 (19.6) | 34 (15.6) | 98 (21.0) | 153 (19.0) | 98 (20.2) | 22 (10.5) |
| 4 | 2812 (19.3) | 26 (11.9) | 73 (15.7) | 128 (15.9) | 94 (19.4) | 23 (11.0) |
| 5 (Most affluent) | 2913 (20.0) | 18 (8.3) | 40 (8.6) | 112 (13.9) | 68 (14.0) | 20 (9.5) |
| Smoking *, n (%) |  |  |  |  |  |  |
| Never | 5027 (34.6) | 46 (21.1) | 123 (26.4) | 220 (27.3) | 141 (29.1) | 91 (43.3) |
| Previous | 3266 (22.5) | 47 (21.6) | 124 (26.6) | 204 (25.3) | 120 (24.7) | 33 (15.7) |
| Current | 2321 (16.0) | 71 (32.6) | 115 (24.7) | 197 (24.5) | 123 (25.4) | 28 (13.3) |
| Missing | 3928 (27.0) | 54 (24.8) | 104 (22.3) | 184 (22.9) | 101 (20.8) | 58 (27.6) |
| Alcohol *, n (%) |  |  |  |  |  |  |
| Never, or up to 2 times/year | 1786 (12.3) | 43 (19.7) | 95 (20.4) | 149 (18.5) | 99 (20.4) | 47 (22.4) |
| Up to 1-2 times/month | 2057 (14.2) | 34 (15.6) | 79 (17.0) | 145 (18.0) | 75 (15.5) | 29 (13.8) |
| 1-4 times/week | 4116 (28.3) | 42 (19.3) | 97 (20.8) | 162 (20.1) | 115 (23.7) | 28 (13.3) |
| Daily or almost daily | 1172 (8.1) | 11 (5.1) | 45 (9.7) | 65 (8.1) | 40 (8.3) | 6 (2.9) |
| Missing | 5411 (37.2) | 88 (40.4) | 150 (32.2) | 284 (35.3) | 156 (32.2) | 100 (47.6) |
| Physical Activity score ^a^, median (IQR) | 5 (0, 20) | 0 (0, 9) | 1 (0, 12) | 3 (0, 15) | 4 (0, 14) | 2 (0, 12) |
| EQ-5D Index Score ^b^, median (IQR) | 0.848 (0.796, 1.00) | 0.656 (0.186, 0.760) | 0.691 (0.291, 0.796) | 0.725 (0.620, 0.848) | 0.727 (0.620, 0.848) | 0.727 (0.656, 0.848) |
| Self-rated health, n (%) |  |  |  |  |  |  |
| Excellent | 3063 (21.1) | 3 (1.4) | 16 (3.4) | 42 (5.2) | 22 (4.5) | 5 (2.4) |
| Very good | 5196 (35.7) | 13 (6) | 52 (11.2) | 125 (15.5) | 69 (14.2) | 18 (8.6) |
| Good | 4203 (28.9) | 34 (15.6) | 95 (20.4) | 207 (25.7) | 130 (26.8) | 66 (31.4) |
| Fair | 1595 (11.0) | 61 (28) | 163 (35) | 229 (28.5) | 148 (30.5) | 65 (31) |
| Poor | 464 (3.2) | 107 (49.1) | 140 (30) | 202 (25.1) | 116 (23.9) | 56 (26.7) |
| Missing | 21 (0.1) | 0 (0) | 0 (0) | 0 (0) | 0 (0) | 0 (0) |

* Data for smoking, alcohol intake and physical activity were not available at baseline and these data were collected at one-year follow up (Wave ‘b’ of UKHLS data collection). ^a^ Continuous count of number of days in past 4 weeks the participant had walked for at least 30 minutes (range 0 – 28). ^b^ EQ-5D index scores calculated from collected SF-12 data

Table S7. Cohort characteristics by MLTC clusters in UKHLS participants aged 55 - 73 years.

|  | **No multimorbidity** | **Pulmonary** | **Hypertension +** | **Cardiovascular** | **Depression, Thyroid & Cancer** |
| --- | --- | --- | --- | --- | --- |
| N (%) | 8191 (66.6) | 730 (5.9) | 2162 (17.6) | 584 (4.7) | 641 (5.2) |
| Female sex, n (%) | 4218 (51.5) | 478 (65.5) | 1198 (55.4) | 226 (38.7) | 436 (68.0) |
| Age (years), median (IQR) | 62 (58, 67) | 63 (59, 68) | 65 (60, 69) | 66 (61, 70) | 63 (59, 67) |
| White ethnicity, n (%) | 7120 (90.5) | 684 (93.7) | 1913 (88.6) | 529 (90.7) | 606 (94.5) |
| Number of LTCs  (from n=13), median (IQR) | 0 (0, 1) | 3 (2, 3) | 2 (2, 3) | 3 (2, 4) | 2 (2, 2) |
| BMI (kg/m^2^), median (IQR) | 25.9 (23.5, 28.7) | 27.0 (24.0, 30.8) | 28.1 (25.2, 31.9) | 27.8 (25.1, 32.3) | 26.7 (23.9, 29.9) |
| Socioeconomic deprivation, n (%) |  |  |  |  |  |
| 1 (Most deprived) | 1258 (15.4) | 152 (20.8) | 476 (22.0) | 177 (30.3) | 113 (17.6) |
| 2 | 1481 (18.1) | 148 (20.3) | 428 (19.8) | 135 (23.1) | 131 (20.4) |
| 3 | 1715 (20.9) | 154 (21.1) | 442 (20.4) | 104 (17.8) | 149 (23.2) |
| 4 | 1784 (21.8) | 144 (19.7) | 448 (20.7) | 86 (14.7) | 144 (22.5) |
| 5 (Most affluent) | 1953 (23.8) | 132 (18.1) | 368 (17.0) | 82 (14.0) | 104 (16.2) |
| Smoking *, n (%) |  |  |  |  |  |
| Never | 2547 (31.1) | 189 (25.9) | 646 (29.9) | 120 (20.6) | 188 (29.3) |
| Previous | 2816 (34.4) | 299 (41.0) | 856 (39.6) | 232 (39.7) | 224 (35.0) |
| Current | 1016 (12.4) | 124 (17.0) | 279 (12.9) | 96 (16.4) | 108 (16.9) |
| Missing | 1812 (22.1) | 118 (16.2) | 381 (17.6) | 136 (23.3) | 121 (18.9) |
| Alcohol *, n (%) |  |  |  |  |  |
| Never, or up to 2 times/year | 1127 (13.8) | 169 (23.2) | 459 (21.2) | 153 (26.2) | 132 (20.6) |
| Up to 1-2 times/month | 1029 (12.6) | 115 (15.8) | 299 (13.8) | 64 (11.0) | 110 (17.2) |
| 1-4 times/week | 2454 (30.0) | 180 (24.7) | 519 (24.0) | 108 (18.5) | 151 (23.6) |
| Daily or almost daily | 1095 (13.4) | 84 (11.5) | 300 (13.9) | 51 (8.7) | 69 (10.8) |
| Missing | 2486 (30.4) | 182 (24.9) | 585 (27.1) | 208 (35.6) | 179 (27.9) |
| Physical Activity score ^a^, median (IQR) | 5 (0, 20) | 2 (0, 12) | 2 (0, 12) | 0 (0, 4) | 3 (0, 14) |
| EQ-5D Index Score ^b^, median (IQR) | 0.848 (0.779, 1.00) | 0.725 (0.620, 0.814) | 0.727 (0.620, 0.848) | 0.656 (0.195, 0.760) | 0.725 (0.620, 0.796) |
| Self-rated health, n (%) |  |  |  |  |  |
| Excellent | 1441 (17.6) | 25 (3.4) | 68 (3.2) | 2 (0.3) | 27 (4.2) |
| Very good | 2771 (33.8) | 115 (15.8) | 310 (14.3) | 41 (7.0) | 105 (16.4) |
| Good | 2378 (29.0) | 193 (26.4) | 667 (30.9) | 84 (14.4) | 185 (28.9) |
| Fair | 1184 (14.5) | 212 (29.0) | 677 (31.3) | 187 (32.0) | 195 (30.4) |
| Poor | 390 (4.8) | 184 (25.2) | 439 (20.3) | 270 (46.2) | 129 (20.1) |
| Missing | 27 (0.3) | 1 (0.1) | 1 (0.1) | 0 (0) | 0 (0) |

* Data for smoking, alcohol intake and physical activity were not available at baseline and these data were collected at one-year follow up (Wave ‘b’ of UKHLS data collection). ^a^ Continuous count of number of days in past 4 weeks the participant had walked for at least 30 minutes (range 0 – 28). ^b^ EQ-5D index scores calculated from collected SF-12 data

Table S8. Cohort characteristics by MLTC clusters in UKHLS participants aged 74+ years.

|  | **No multimorbidity** | **Cardiometabolic** | **Pulmonary** | **Arthritis +** |
| --- | --- | --- | --- | --- |
| N (%) | 2161 (53.4) | 547 (13.5) | 380 (9.4) | 959 (23.7) |
| Female sex, n (%) | 1131 (52.3) | 230 (42.1) | 232 (61.1) | 633 (66.0) |
| Age (years), median (IQR) | 79 (74, 83) | 79 (76, 83) | 79 (76, 82) | 79 (76, 83) |
| White ethnicity, n (%) | 1900 (94.0) | 510 (93.2) | 356 (93.7) | 901 (94) |
| Number of LTCs  (from n=13), median (IQR) | 1 (0, 1) | 2 (2, 3) | 3 (2, 4) | 3 (2, 3) |
| BMI (kg/m^2^), median (IQR) | 24.7 (22.5, 27.7) | 25.8 (23.3, 28.7) | 26.1 (23.1, 29.4) | 26.3 (23.5, 29.7) |
| Socioeconomic deprivation, n (%) |  |  |  |  |
| 1 (Most deprived) | 341 (15.8) | 108 (19.7) | 85 (22.4) | 176 (18.4) |
| 2 | 411 (19.0) | 96 (17.6) | 79 (20.8) | 176 (18.4) |
| 3 | 456 (21.1) | 113 (20.7) | 69 (18.2) | 223 (23.3) |
| 4 | 466 (21.6) | 110 (20.1) | 72 (19.0) | 196 (20.4) |
| 5 (Most affluent) | 487 (22.5) | 120 (21.9) | 75 (19.7) | 188 (19.6) |
| Smoking *, n (%) |  |  |  |  |
| Never | 653 (30.2) | 155 (28.3) | 94 (24.7) | 304 (31.7) |
| Previous | 752 (34.8) | 237 (43.3) | 169 (44.5) | 371 (38.7) |
| Current | 110 (5.1) | 22 (4.0) | 27 (7.1) | 46 (4.8) |
| Missing | 646 (29.9) | 133 (24.3) | 90 (23.7) | 238 (24.8) |
| Alcohol *, n (%) |  |  |  |  |
| Never, or up to 2 times/year | 388 (18.0) | 119 (21.8) | 98 (25.8) | 234 (24.4) |
| Up to 1-2 times/month | 242 (11.2) | 75 (13.7) | 50 (13.2) | 114 (11.9) |
| 1-4 times/week | 394 (18.2) | 87 (15.9) | 49 (12.9) | 150 (15.6) |
| Daily or almost daily | 228 (10.6) | 55 (10.1) | 38 (10.0) | 96 (10.0) |
| Missing | 909 (42.1) | 211 (38.6) | 145 (38.2) | 365 (38.1) |
| Physical Activity score *^a^, median (IQR) | 1 (0, 9) | 0 (0, 5) | 0 (0, 2) | 0 (0, 2) |
| EQ-5D Index Score ^b^, median (IQR) | 0.796 (0.692, 1) | 0.727 (0.656, 0.848) | 0.691 (0.228, 0.796) | 0.691 (0.585, 0.796) |
| Self-rated health, n (%) |  |  |  |  |
| Excellent | 261 (12.1) | 14 (2.6) | 6 (1.6) | 31 (3.2) |
| Very good | 579 (26.8) | 80 (14.6) | 41 (10.8) | 140 (14.6) |
| Good | 636 (29.4) | 150 (27.4) | 63 (16.6) | 217 (22.6) |
| Fair | 438 (20.3) | 189 (34.6) | 129 (34.0) | 327 (34.1) |
| Poor | 235 (10.9) | 112 (20.5) | 141 (37.1) | 243 (25.4) |
| Missing | 12 (0.6) | 2 (0.4) | 0 (0) | 1 (0.1) |

* Data for smoking, alcohol intake and physical activity were not available at baseline and these data were collected at first follow up (Wave ‘b’ of UKHLS data collection). ^a^ Continuous count of number of days in past 4 weeks the participant had walked for at least 30 minutes (range 0 – 28). ^b^ EQ-5D index scores calculated from collected SF-12 data.

Table S9. Comparison of baseline cohort characteristics in participants with and without HRQoL follow up data in UK Biobank

|  | **HRQoL Follow up data?** | |
| --- | --- | --- |
|  | **Yes (n = 168,185)** | **No (n = 335,178)** |
| Age (years), median (IQR) | 57 (50, 62) | 58 (50, 64) |
| Age category, n (%) |  |  |
| 37 – 54 years | 69,714 (41.7) | 124,395 (37.1) |
| 55 – 73 years | 97,471 (58.3) | 210,783 (62.9) |
| Female sex, n (%) | 94,988 (56.8) | 178,311 (53.2) |
| White ethnicity, n (%) | 161,720 (96.7) | 310,849 (92.7) |
| Socioeconomic deprivation ^a^, n (%) |  |  |
| Quintile 1 (Most affluent) | 77,920 (46.6) | 135,168 (40.3) |
| 2 | 23,192 (13.9) | 44,528 (13.3) |
| 3 | 22,187 (13.3) | 43,629 (13.0) |
| 4 | 24,444 (14.6) | 53,407 (15.9) |
| Quintile 5 (Most deprived) | 19,255 (11.5) | 58,010 (17.3) |
| Missing | 187 (0.1) | 436 (0.1) |
| Number of long-term conditions, n (%) |  |  |
| 0 LTCs | 65,001 (38.9) | 108,071 (32.2) |
| 1 LTC | 56,315 (33.7) | 107,853 (32.2) |
| 2 LTCs | 28,764 (17.2) | 66,763 (19.9) |
| 3 LTCs | 11,351 (6.8) | 31,969 (9.5) |
| ≥4 LTCs | 5754 (3.4) | 20,522 (6.1) |
| BMI (kg/m^2^) | 26.1 (23.6, 29.1) | 27.1 (24.4, 30.3) |
| Alcohol intake (weekly units), median (IQR) | 10 (2.5, 20.5) | 9 (0, 21) |
| Alcohol frequency, n (%) |  |  |
| Never | 9181 (5.5) | 31,443 (9.4) |
| Special occasions only | 15,419 (9.2) | 42,570 (12.7) |
| 1-3 times per month | 18,386 (11.0) | 37,450 (11.2) |
| 1-2 times per week | 42,054 (25.2) | 87,203 (26.0) |
| 3-4 times per week | 43,613 (26.1) | 71,800 (21.4) |
| Daily/almost daily | 38,400 (23.0) | 63,344 (18.9) |
| Missing | 132 (0.1) | 1368 (0.4) |
| Smoking, n (%) |  |  |
| Never | 96,584 (57.8) | 176,864 (52.8) |
| Previous | 58,306 (34.9) | 114,702 (34.2) |
| Current | 11,882 (7.1) | 41,077 (12.3) |
| Missing | 413 (0.3) | 2535 (0.8) |
| Physical activity, n (%) |  |  |
| High | 21,395 (12.8) | 28,660 (8.6) |
| Moderate | 133,121 (79.6) | 260,268 (77.7) |
| Low | 4752 (2.8) | 18,181 (4.2) |
| None | 6572 (3.9) | 26,265 (7.8) |
| Missing | 1345 (0.8) | 5804 (1.7) |
| Frailty phenotype ^b^, n (%) |  |  |
| Robust | 107,565 (64.3) | 180,475 (53.8) |
| Pre-frail | 54,582 (32.7) | 130,593 (39.0) |
| Frail | 2797 (1.7) | 14,765 (4.4) |
| Missing | 2241 (1.3) | 9345 (2.8) |
| Self-rated health, n (%) |  |  |
| Excellent | 36,547 (21.9) | 45,285 (13.5) |
| Good | 99,991 (59.8) | 188,955 (56.4) |
| Fair | 26,186 (15.7) | 79,146 (23.6) |
| Poor | 4077 (2.4) | 18,691 (5.6) |
| Missing | 384 (0.2) | 3101 (0.9) |

^a^ Using UK population distribution of Townsend deprivation scores from 2001 Census data. ^b^ Freid frailty phenotype.

Table S10. Comparison of baseline cohort characteristics in participants with and without HRQoL follow up data in UKHLS.

|  | **HRQoL Follow up data?** | |
| --- | --- | --- |
|  | **Yes (n = 21,837)** | **No (n = 27,349)** |
| Age (years), median (IQR) | 47 (36, 60) | 43 (30, 60) |
| Age category, n (%) |  |  |
| 18 – 36 years | 5903 (27.0) | 10,202 (37.3) |
| 37 – 54 years | 8208 (37.6) | 8518 (31.2) |
| 55 – 73 years | 6454 (29.6) | 5854 (21.4) |
| 74+ years | 1272 (5.8) | 2775 (10.2) |
| Female sex, n (%) | 12,453 (57.0) | 14,404 (52.7) |
| White ethnicity, n (%) |  |  |
| Socioeconomic deprivation ^a^, n (%) |  |  |
| Quintile 1 (Most affluent) | 4303 (19.7) | 7159 (26.2) |
| 2 | 4242 (19.4) | 6041 (22.1) |
| 3 | 4479 (20.5) | 5180 (18.9) |
| 4 | 4387 (20.1) | 4670 (17.1) |
| Quintile 5 (Most deprived) | 4426 (20.3) | 4299 (15.7) |
| Missing | 0 (0) | 0 (0) |
| Number of long-term conditions, n (%) |  |  |
| 0 LTCs | 11,361 (52.0) | 15,930 (58.3) |
| 1 LTC | 6465 (29.6) | 6554 (24.0) |
| 2 LTCs | 2499 (11.4) | 2690 (9.8) |
| 3 LTCs | 966 (4.4) | 1305 (4.8) |
| ≥4 LTCs | 546 (2.5) | 870 (3.2) |
| BMI (kg/m^2^), median (IQR) | 25.7 (23.0, 29.0) | 25.3 (22.4, 28.5) |
| Alcohol frequency *, n (%) |  |  |
| Never, or up to 2 times/year | 3777 (17.3) | 3364 (12.3) |
| Up to 1-2 times/month | 4264 (19.5) | 2701 (9.9) |
| 1-4 times/week | 7535 (34.5) | 4463 (16.3) |
| Daily or almost daily | 2503 (11.5) | 1310 (4.8) |
| Missing | 3758 (17.2) | 15,511 (56.7) |
| Smoking *, n (%) |  |  |
| Never | 8661 (39.7) | 6524 (23.9) |
| Previous | 7723 (25.4) | 4728 (17.3) |
| Current | 3993 (18.3) | 3397 (12.4) |
| Missing | 1460 (6.7) | 12,700 (46.4) |
| Physical activity score *^a^, median (IQR) | 4 (0, 16) | 4 (0, 15) |
| EQ-5D Index Score ^a^, median (IQR) | 0.848 (0.727, 1) | 0.848 (0.725, 1) |
| Self-rated health, n (%) |  |  |
| Excellent | 3981 (18.2) | 5034 (18.4) |
| Very good | 7354 (33.7) | 8270 (30.2) |
| Good | 6207 (28.4) | 7379 (27.0) |
| Fair | 3047 (14.0) | 4147 (15.2) |
| Poor | 1234 (5.7) | 2440 (8.9) |
| Missing | 14 (0.1) | 79 (0.3) |

* Data for smoking, alcohol intake and physical activity were not available at baseline and these data were collected at first follow up (Wave ‘b’ of UKHLS data collection). ^a^ Continuous count of number of days in past 4 weeks the participant had walked for at least 30 minutes (range 0 – 28). ^b^ EQ-5D index scores calculated from collected SF-12 data.

Table S11. Sensitivity analysis: Summary of MLTC clusters when socioeconomic status included as a variable in LCA models

| **Potential Cluster Label** | **Leading LTCs (within-cluster prevalence [%])** | **N (%) ^a^** | **No. of LTCs ^b^, median (IQR)** | **Female (%)** | **Most deprived ^c^ (%)** | **Predicted probability of cluster membership, mean (SD)** |
| --- | --- | --- | --- | --- | --- | --- |
|  | ***18 – 36 years*** |  |  |  |  |  |
|  | **UKHLS (n = 16,105)** |  |  |  |  |  |
| No multimorbidity |  | 15,416 (95.7) | 0 (0, 0) | 55 | 30 |  |
| *Pulmonary* | **Asthma** (100%), Hypertension (33%), Arthritis (23%), **COPD** (14%) | 265 (1.7) | 2 (2, 2) | 65 | 29 | 0.94 (0.07) |
| *Cardiometabolic* | Depression (59%), **Hypertension** (55%), **Diabetes** (23%), Arthritis (21%) | 255 (1.6) | 2 (2, 2) | 70 | 36 | 0.99 (0.07) |
| *Depression & Asthma* | **Depression** (100%), Asthma (100%) | 169 (1.0) | 2 (2, 3) | 74 | 39 | 0.86 (0.13) |
|  |  |  |  |  |  |  |
|  | ***37 – 54 years*** |  |  |  |  |  |
|  | **UK BIOBANK (n = 194,109)** |  |  |  |  |  |
| No multimorbidity |  | 151,975 (78) | 0 (0, 1) | 55 | 17 |  |
| *Hypertension +* | **Hypertension** (100%), Painful conditions (27%), Asthma (19%), Diabetes (17%) | 14,577 (7.5) | 2 (2, 3) | 49 | 18 | 0.78 (0.17) |
| *Pain, Thyroid & Cancer* | **Painful conditions** (45%), Dyspepsia (24%), **Migraine** (19%), **Thyroid** **disease** (20%), **Cancer** (18%) | 9862 (5.1) | 2 (2, 3) | 69 | 18 | 0.95 (0.09) |
| *Asthma +* | **Asthma** (100%), Painful conditions (24%), **Psoriasis/Eczema** (20%) | 8492 (4.4) | 2 (2, 3) | 63 | 18 | 0.93 (0.13) |
| *Depression & Anxiety* | **Depression** (100%), Painful conditions (30%), **Anxiety** (18%) | 6313 (3.3) | 2 (2, 3) | 70 | 30 | 0.79 (0.18) |
| *Cardiometabolic* | **Hypertension** (63%), **Diabetes** (32%), Painful conditions (26%), **Coronary heart disease** (23%), **Stroke** (10%) | 2806 (1.4) | 3 (2, 3) | 37 | 75 | 0.71 (0.16) |
|  |  |  |  |  |  |  |
|  | **UKHLS (n = 16,726)** |  |  |  |  |  |
| No multimorbidity |  | 14,542 (86.9) | 0 (0, 1) | 53 | 21 |  |
| *Pulmonary* | **Asthma** (100%), Depression (36%), Hypertension (35%), **COPD** (18%) | 809 (4.8) | 2 (2, 3) | 68 | 30 | 0.99 (0.05) |
| *Depression, Thyroid & Cancer* | Hypertension (62%), **Depression** (58%), **Thyroid disease** (25%), **Cancer** (20%) | 491 (2.9) | 2 (2, 3) | 65 | 24 | 0.98 (0.07) |
| *Arthritis +* | **Arthritis** (100%), Hypertension (50%), Depression (38%) | 476 (2.8) | 2 (2, 3) | 67 | 32 | 0.99 (0.07) |
| *Cardiovascular* | **CHD** (100%), Hypertension (57%), Arthritis (38%), Asthma (26%), Diabetes (21%), **Stroke** (11%) | 212 (1.3) | 3 (2, 4) | 45 | 43 | 0.91 (0.10) |
| *Hypertension & Diabetes* | **Hypertension** (100%), **Diabetes** (100%) | 196 (1.2) | 2 (2, 3) | 52 | 48 | 0.84 (0.14) |
|  |  |  |  |  |  |  |
|  | ***55 – 73 years*** |  |  |  |  |  |
|  | **UK BIOBANK (n = 308,254)** |  |  |  |  |  |
| No multimorbidity |  | 185,265 (60) | 1 (0, 1) | 53 | 12 |  |
| *Cardiometabolic* | **Hypertension** (87%), Painful conditions (33%), **Diabetes** (29%), Coronary heart disease (24%) | 51,267 (17) | 2 (2, 3) | 41 | 20 | 0.80 (0.15) |
| *Pain +* | **Painful** **conditions** (51%), Hypertension (37%), **Dyspepsia** (28%), Thyroid disease (18%) | 34,711 (11) | 2 (2, 3) | 66 | 14 | 0.84 (0.16) |
| *Pulmonary* | **Asthma** (100%), Painful conditions (24%), Psoriasis/Eczema (19%), **COPD** (12%) | 20,538 (6.7) | 2 (2, 3) | 61 | 15 | 0.69 (0.15) |
| *Cancer +* | **Cancer** (100%), Hypertension (44%), | 16,340 (5.3) | 2 (2, 3) | 62 | 13 | 0.79 (0.12) |
|  |  |  |  |  |  |  |
|  | **UKHLS (n = 12,308)** |  |  |  |  |  |
| No multimorbidity |  | 8191 (66.6) | 0 (0, 1) | 52 | 15 |  |
| *Hypertension +* | **Hypertension** (100%), Arthritis (54%), Diabetes (31%) | 2163 (17.6) | 2 (2, 3) | 55 | 21 | 0.88 (0.12) |
| *Pulmonary* | **Asthma** (100%), Arthritis (57%), Hypertension (35%), **COPD** (32%) | 748 (6.1) | 3 (2, 3) | 65 | 20 | 0.80 (0.19) |
| *Depression, Thyroid & Cancer* | Arthritis (75%), **Depression** (30%), **Thyroid disease** (29%), **Cancer** (28%) | 672 (5.5) | 2 (2, 2) | 67 | 16 | 0.89 (0.13) |
| *Cardiovascular* | **CHD** (72%), Hypertension (51%), Arthritis (51%), Diabetes (35%), Asthma (24%), **Stroke** (22%), Heart failure (17%) | 534 (4.3) | 3 (2, 5) | 38 | 38 | 0.78 (0.17) |
|  |  |  |  |  |  |  |
|  | ***74+ years*** |  |  |  |  |  |
|  | **UKHLS (n = 4047)** |  |  |  |  |  |
| No multimorbidity |  | 2161 (53.4) | 1 (0, 1) | 52 | 16 |  |
| *Arthritis +* | **Arthritis** (100%)**,** Hypertension (70%), CHD (31%) | 959 (23.7) | 3 (2, 3) | 66 | 18 | 1.00 (0.00) |
| *Cardiometabolic* | Hypertension (78%), **CHD** (49%), **Diabetes** (40%) **Stroke** (22%) | 547 (13.5) | 2 (2, 3) | 42 | 20 | 1.00 (0.00) |
| *Pulmonary* | **Asthma** (100%), Arthritis (59%), Hypertension (55%), CHD (29%), **COPD** (24%) | 380 (9.4) | 3 (2, 4) | 61 | 22 | 1.00 (0.00) |

Table S12. Association between MLTC and HRQoL at 10-years follow up in UK Biobank: combined model with MLTC clusters and LTC counts.

|  | **Model 3: Combined** | | |
| --- | --- | --- | --- |
|  | **β (95% CI)** | **Std. Error** | **p-value** |
| ***37 – 54 years*** |  |  |  |
| No multimorbidity | Ref. |  |  |
|  |  |  |  |
| *Hypertension & Diabetes* | 0.024 (0.015, 0.034) | 0.005 | <0.001 |
| *Asthma +* | 0.005 (-0.004, 0.015) | 0.004 | 0.3 |
| *Thyroid & Cancer* | 0.016 (0.005, 0.027) | 0.006 | 0.01 |
| *Depression & Anxiety* | -0.034 (-0.046, -0.021) | 0.006 | <0.001 |
| *Pain +* | -0.034 (-0.045, -0.022) | 0.006 | <0.001 |
|  |  |  |  |
| LTC count (continuous) | -0.031 (-0.046, -0.021) | 0.006 | <0.001 |
|  |  |  |  |
| ***55 – 73 years*** |  |  |  |
| No multimorbidity | Ref. |  |  |
|  |  |  |  |
| *Cardiometabolic* | 0.007 (0.001, 0.012) | 0.003 | 0.02 |
| *Pain +* | -0.023 (-0.029, -0.017) | 0.003 | <0.001 |
| *Pulmonary* | 0.006 (-0.001, 0.013) | 0.004 | 0.07 |
| *Cancer +* | 0.013 (0.006, 0.02) | 0.004 | 0.001 |
|  |  |  |  |
| LTC count (continuous) | -0.024 (-0.026, -0.021) | 0.001 | <0.001 |

Covariates: age, sex, ethnicity, deprivation (Townsend quintile), BMI, smoking, alcohol frequency, alcohol weekly units, physical activity, frailty phenotype, and baseline self-rated health. ‘No multimorbidity’ were the reference group in all models.

Table S13. Association between MLTC and HRQoL at 5-years follow up in UKHLS: combined model with MLTC clusters and LTC counts.

|  | **Model 3: Combined** | | |
| --- | --- | --- | --- |
|  | **β (95% CI)** | **Std. Error** | **p-value** |
| ***18 – 36 years*** |  |  |  |
| No multimorbidity | Ref. |  |  |
|  |  |  |  |
| *Pulmonary* | 0.03 (-0.039, 0.099) | 0.035 | 0.4 |
| *Hypertension & Diabetes* | -0.017 (-0.094, 0.061) | 0.04 | 0.68 |
| *Depression & Asthma* | 0.005 (-0.062, 0.072) | 0.034 | 0.89 |
|  |  |  |  |
| LTC count (continuous) | -0.059 (-0.079, -0.039) | 0.01 | <0.001 |
|  |  |  |  |
| ***37 – 54 years*** |  |  |  |
| No multimorbidity | Ref. |  |  |
|  |  |  |  |
| *Pulmonary* | -0.011 (-0.057, 0.034) | 0.023 | 0.64 |
| *Depression, Thyroid & Cancer* | 0.029 (-0.022, 0.082) | 0.027 | 0.27 |
| *Arthritis +* | -0.062 (-0.117, -0.007) | 0.028 | 0.03 |
| *Cardiometabolic* | -0.122 (-0.217, -0.026) | 0.049 | 0.01 |
| *Hypertension & Diabetes* | -0.007 (-0.09, 0.076) | 0.043 | 0.87 |
|  |  |  |  |
| LTC count (continuous) | -0.056 (-0.072, -0.041) | 0.008 | <0.001 |
|  |  |  |  |
| ***55 – 73 years*** |  |  |  |
| No multimorbidity | Ref. |  |  |
|  |  |  |  |
| *Hypertension +* | 0.002 (-0.028, 0.032) | 0.016 | 0.9 |
| *Pulmonary* | -0.006 (-0.045, 0.034) | 0.02 | 0.78 |
| *Arthritis, Depression & Cancer* | -0.023 (-0.062, 0.017) | 0.02 | 0.26 |
| *Cardiometabolic* | -0.035 (-0.082, 0.013) | 0.024 | 0.15 |
|  |  |  |  |
| LTC count (continuous) | -0.038 (-0.05, -0.02) | 0.006 | <0.001 |
|  |  |  |  |
| ***74+ years*** |  |  |  |
| No multimorbidity | Ref. |  |  |
|  |  |  |  |
| *Arthritis +* | -0.058 (-0.123, 0.006) | 0.033 | 0.08 |
| *Cardiometabolic* | -0.041 (-0.11, 0.027) | 0.035 | 0.24 |
| *Pulmonary* | -0.001 (-0.092, 0.09) | 0.046 | 0.99 |
|  |  |  |  |
| LTC count (continuous) | -0.019 (-0.044, 0.007) | 0.013 | 0.15 |

Covariates: age, sex, ethnicity, deprivation (IMD quintile), BMI, smoking, alcohol frequency, physical activity, and baseline EQ-5D index score. ‘No multimorbidity’ were the reference group in all models.

Table S14. Sensitivity analyses: OLS regression models for EQ-5D Index at 10-years follow up in UK Biobank.

|  | **Model 1: LTC Counts** | | | **Model 2: MLTC Clusters** | | |
| --- | --- | --- | --- | --- | --- | --- |
|  | **β (95% CI)** | **Std. Error** | **p-value** | **β (95% CI)** | **Std. Error** | **p-value** |
| ***37 – 54 years*** |  |  |  |  |  |  |
| No multimorbidity | Ref. |  |  | Ref. |  |  |
| 2 LTCs | -0.031 (-0.035, -0.027) | 0.002 | <0.001 |  |  |  |
| 3 LTCs | -0.058 (-0.065, -0.051) | 0.004 | <0.001 |  |  |  |
| ≥4 LTCs | -0.101 (-0.113, -0.09) | 0.006 | <0.001 |  |  |  |
|  |  |  |  |  |  |  |
| *Hypertension & Diabetes* |  |  |  | -0.026 (-0.032, -0.021) | 0.003 | <0.001 |
| *Asthma +* |  |  |  | -0.042 (-0.048, -0.036) | 0.003 | <0.001 |
| *Thyroid & Cancer* |  |  |  | -0.025 (-0.032, -0.018) | 0.004 | <0.001 |
| *Depression & Anxiety* |  |  |  | -0.081 (-0.09, -0.071) | 0.005 | <0.001 |
| *Pain +* |  |  |  | -0.07 (-0.078, -0.061) | 0.004 | <0.001 |
|  |  |  |  |  |  |  |
| ***55 – 73 years*** |  |  |  |  |  |  |
| No multimorbidity | Ref. |  |  | Ref. |  |  |
| 2 LTCs | -0.025 (-0.027, -0.022) | 0.001 | <0.001 |  |  |  |
| 3 LTCs | -0.043 (-0.047, -0.039) | 0.002 | <0.001 |  |  |  |
| ≥4 LTCs | -0.073 (-0.079, -0.067) | 0.003 | <0.001 |  |  |  |
|  |  |  |  |  |  |  |
| *Cardiometabolic* |  |  |  | -0.027 (-0.03, -0.024) | 0.002 | <0.001 |
| *Pain +* |  |  |  | -0.052 (-0.055, -0.048) | 0.002 | <0.001 |
| *Pulmonary* |  |  |  | -0.029 (-0.033, -0.025) | 0.002 | <0.001 |
| *Cancer +* |  |  |  | -0.022 (-0.027, -0.017) | 0.002 | <0.001 |

Covariates: age, sex, ethnicity, deprivation (Townsend quintile), BMI, smoking, alcohol frequency, alcohol weekly units, physical activity, frailty phenotype, and baseline self-rated health. ‘No multimorbidity’ were the reference group in all models.

Table S15. Sensitivity analyses: OLS regression results for EQ-5D Index scores at median 5-year follow up in UKHLS.

|  | **Model 1: LTC Counts** | | | **Model 2: MLTC Clusters** | | |
| --- | --- | --- | --- | --- | --- | --- |
|  | **β (95% CI)** | **Std. Error** | **p-value** | **β (95% CI)** | **Std. Error** | **p-value** |
| ***18 – 36 years*** |  |  |  |  |  |  |
| No multimorbidity | Ref. |  |  | Ref. |  |  |
| 2 LTCs | -0.06 (-0.092, -0.028) | 0.016 | <0.001 |  |  |  |
| 3 LTCs | -0.133 (-0.202, -0.065) | 0.035 | <0.001 |  |  |  |
| ≥4 LTCs | -0.148 (-0.461, 0.164) | 0.159 | 0.352 |  |  |  |
|  |  |  |  |  |  |  |
| *Pulmonary* |  |  |  | -0.058 (-0.101, -0.015) | 0.022 | 0.009 |
| *Hypertension & Diabetes* |  |  |  | -0.103 (-0.161, -0.046) | 0.029 | <0.001 |
| *Depression & Asthma* |  |  |  | -0.06 (-0.103, -0.018) | 0.022 | 0.005 |
|  |  |  |  |  |  |  |
| ***37 – 54 years*** |  |  |  |  |  |  |
| No multimorbidity | Ref. |  |  | Ref. |  |  |
| 2 LTCs | -0.077 (-0.1, -0.055) | 0.012 | <0.001 |  |  |  |
| 3 LTCs | -0.126 (-0.167, -0.085) | 0.021 | <0.001 |  |  |  |
| ≥4 LTCs | -0.258 (-0.323, -0.192) | 0.033 | <0.001 |  |  |  |
|  |  |  |  |  |  |  |
| *Pulmonary* |  |  |  | -0.094 (-0.124, -0.065) | 0.015 | <0.001 |
| *Depression, Thyroid & Cancer* |  |  |  | -0.051 (-0.087, -0.015) | 60.018 | 0.005 |
| *Arthritis +* |  |  |  | -0.137 (-0.178, -0.096) | 0.021 | <0.001 |
| *Cardiometabolic* |  |  |  | -0.252 (-0.33, -0.173) | 0.04 | <0.001 |
| *Hypertension & Diabetes* |  |  |  | -0.081 (-0.149, -0.013) | 0.035 | 0.019 |
|  |  |  |  |  |  |  |
| ***55 – 73 years*** |  |  |  |  |  |  |
| No multimorbidity | Ref. |  |  | Ref. |  |  |
| 2 LTCs | -0.038 (-0.052, -0.023) | 0.007 | <0.001 |  |  |  |
| 3 LTCs | -0.067 (-0.091, -0.042) | 0.013 | <0.001 |  |  |  |
| ≥4 LTCs | -0.159 (-0.185, -0.123) | 0.018 | <0.001 |  |  |  |
|  |  |  |  |  |  |  |
| *Hypertension +* |  |  |  | -0.047 (-0.063, -0.031) | 0.008 | <0.001 |
| *Pulmonary* |  |  |  | -0.062 (-0.088, -0.036) | 0.013 | <0.001 |
| *Arthritis, Depression & Cancer* |  |  |  | -0.056 (-0.085, -0.027) | 0.015 | <0.001 |
| *Cardiometabolic* |  |  |  | -0.107 (-0.143, -0.07) | 0.019 | <0.001 |
|  |  |  |  |  |  |  |
| ***74+ years*** |  |  |  |  |  |  |
| No multimorbidity | Ref. |  |  | Ref. |  |  |
| 2 LTCs | -0.05 (-0.084, -0.016) | 0.017 | 0.004 |  |  |  |
| 3 LTCs | -0.075 (-0.123, -0.028) | 0.024 | 0.002 |  |  |  |
| ≥4 LTCs | -0.069 (-0.131, -0.006) | 0.032 | 0.032 |  |  |  |
|  |  |  |  |  |  |  |
| *Arthritis +* |  |  |  | -0.071 (-0.108, -0.034) | 0.019 | <0.001 |
| *Cardiometabolic* |  |  |  | -0.056 (-0.1, -0.013) | 0.022 | 0.01 |
| *Pulmonary* |  |  |  | -0.03 (-0.087, 0.028) | 0.029 | 0.312 |

Covariates: age, sex, ethnicity, deprivation (IMD quintile), BMI, smoking, alcohol frequency, physical activity, and baseline EQ-5D Index. ‘No multimorbidity’ were the reference group in all models.

UK Biobank Baseline participants

n = 502,640 (100%)

Email invitation for online follow up (only participants who provided valid email address invited ^a^):

n = 335,587 (66.8%)

Completed online follow up with HRQoL data ^b^: n = 167,185 (33.3%)

Excluded, consent withdrawn:

n = 277 (0.1%)

Included in HRQoL regression analyses:

n = 163,121 (32.5%)

Excluded, missing covariates:

n = 4064 (0.8%)

n =

Included in LCA modelling:

n = 502,363 (99.9%)

Lost to follow up (no attempt, incomplete or incorrect data, or died before follow up): n = 168,402 (33.5%)

Not invited for online follow up, no email address: n = 166,776 (33.2%)

n = 335,179

Figure S1. Flow diagram of participant inclusion in UK Biobank analyses.

^a^ Participants without an email address could still access and complete online follow up via the UK Biobank participant website, and information was provided about the follow up in participant newsletters (see: <https://biobank.ndph.ox.ac.uk/showcase/refer.cgi?id=2718>) ^b^ Includes n = 495 participants who did not receive email invitation but accessed and completed the online follow up through the UK Biobank participant website.

Figure S2. Flow diagram of participant inclusion in UKHLS analyses.

UKHLS Baseline participants

(aged ≥18years) included in LCA modelling: n = 49,186 (100%)

HRQoL data at 5-year follow up:

n = 21,162 (43%)

Included in HRQoL regression analyses: n = 16,819 (34.2%)

Excluded, missing covariates:

n = 4343 (8.8%)

HRQoL data at baseline:

n = 45,774 (93.1%)

Lost to follow up:

n = 24,612 (50%)

Excluded, missing HRQoL data:

n = 3412 (6.9%)

n = 24,612 (50%)
